# Single-cell RNA sequencing identifies the prolactin receptor as a therapeutic target in adenomyosis

**DOI:** 10.1038/s41392-025-02339-z

**Published:** 2025-08-13

**Authors:** Runze Wang, Shuai Xu, Qionghua Cui, Xin Chen, Xuelian Wang, Jinghao Liu, Jie Liu, Yuxuan Xin, Hao Shen, Fengxiang Lv, Lan Zhu, Xinli Hu, Rui-Ping Xiao

**Affiliations:** 1 Peking-Tsinghua Center for Life Sciences, Peking University, Beijing, China; 2 Institute of Molecular Medicine, College of Future Technology, Peking University, Beijing, China; 3 Department of Obstetrics and Gynecology, Peking Union Medical College Hospital, Beijing, China; 4 Dazhou Central Hospital, Dazhou, Sichuan China; 5 State Key Laboratory of Membrane Biology, Peking University, Beijing, China; 6 Beijing City Key Laboratory of Cardiometabolic Molecular Medicine, Peking University, Beijing, China; 7 PKU-Nanjing Joint Institute of Translational Medicine, Nanjing, China

**Keywords:** Molecular medicine, Reproductive disorders

## Abstract

Adenomyosis is a complex gynecological disease characterized by endometrial tissue invasion into the myometrium. Current interventions, such as hormonal therapy or hysterectomy, are associated with significant side effects and compromise fertility, underscoring the urgent need for safe and effective treatments. Using single-cell RNA sequencing (scRNA-seq) of uterine samples from patients, we identified prolactin (PRL) signaling as a key pathological driver of adenomyosis. Specifically, scRNA-seq revealed a distinct epithelial subcluster with enriched PRL receptor (PRLR) expression. PRL signaling is overactivated in this epithelial subcluster, promoting cellular survival and proliferation, which contributes to lesion formation and expansion in adenomyosis. Concurrently, PRLR is also highly expressed in a fibroblast subcluster characterized by strong expression of inflammation-related genes. Pathological PRL hyperactivation was further validated in preclinical animal models, where transgenic overexpression of PRL or pituitary transplantation induced an adenomyosis phenotype. Importantly, we demonstrated that dysregulation of local PRL signaling led to the development and progression of adenomyosis, whereas inhibition of PRLR with the monoclonal antibody HMI-115 markedly ameliorated pathological manifestations. These findings establish PRL signaling as a critical driver of adenomyosis pathogenesis, highlighting PRLR inhibition as a promising therapeutic strategy and demonstrating the translational potential of HMI-115 for treating adenomyosis, a gynecological condition that has long been neglected in drug development.

## Introduction

Adenomyosis is characterized by the invasion of endometrial tissue into the myometrium^1^ and is associated with the proliferation and hypertrophy of surrounding myometrial cells, leading to thickening of the uterine wall and enlargement of the whole uterus.^2^ The clinical manifestations of adenomyosis include dysmenorrhea, heavy menstrual bleeding, chronic pelvic pain, and infertility,^3,4^ significantly impairing women’s quality of life. Although its estimated prevalence varies due to inconsistency in diagnostic criteria, adenomyosis is increasingly recognized as a major gynecological condition affecting tens to hundreds of millions of women of reproductive age,^5,6^ and therefore, greater awareness and investigation are needed.^7^

Current drugs for adenomyosis include nonsteroidal anti-inflammatory drugs (NSAIDs), hormonal contraceptives (including combined estrogen-progesterone or progestin-only formulations), and gonadotropin-releasing hormone (GnRH) agonists or antagonists. NSAIDs are used mainly for pain relief and carry risks of gastrointestinal and neurological adverse effects with prolonged use. Treatments with contraceptives frequently induce hypoestrogenic side effects, such as hot flashes and bone mineral density loss. Moreover, hyperestrogenism and the resulting progesterone resistance play critical roles in the pathogenesis of adenomyosis.^8^ Thus, patients display reduced expression of progesterone receptors^9^ and may be unresponsive to progestin treatment. The use of GnRH agonists and antagonists is still based on the mechanism of estrogen suppression; therefore, they are often associated with irreversible osteoporosis, depression, and acne.^10–12^ Even with add-back therapy to address hypoestrogenism caused by GnRH antagonism, the dosing regimens used to titrate estrogen levels are complex and time-consuming. Most importantly, owing to their anti-gonadotrophic effects, these drugs inhibit ovulation, limiting their suitability for women seeking to conceive. Surgical removal of lesions has high recurrence rates,^13,14^ and hysterectomy is not an option for women wishing to preserve fertility. Both procedures also carry inherent surgical risks. Despite the significant adverse effects associated with currently available therapeutic strategies, the development of adenomyosis treatment has stagnated for decades, with no novel molecular targets advancing to clinical trials. Therefore, there is an urgent need for therapies that address adenomyosis pathogenesis without compromising endocrine or reproductive function.

The lack of safe and effective fertility-compatible treatments is due mainly to the limited understanding of the etiology of adenomyosis. One hypothesis suggests that lesions arise from the invagination of basal endometrial tissue, exacerbated by factors such as rapid uterine peristalsis or surgical interventions that disrupt the junctional zone between the endometrium and myometrium, leading to eutopic metaplasia during frequent tissue injury and repair (TIAR).^15,16^ Another theory proposes that adenomyosis results from the proliferation and differentiation of residual embryonic Müllerian ducts or adult stem cells within the myometrium.^17^ However, both hypotheses lack substantial evidence, and the mechanisms underlying ectopic endometrial cell proliferation and lesion spread remain poorly understood.

In this study, we employed single-cell RNA sequencing (scRNA-seq) of endometrial tissues from adenomyosis patients to map the transcriptome profiles of eutopic and ectopic cells. Comparative analysis with non-adenomyosis subjects revealed significant changes in endometrial cell composition, characteristics, and intercellular communication, which may contribute to the clinical symptoms of adenomyosis. By further demarcating cells into subclusters and investigating their changes under pathological conditions, we identified hyperactivation of prolactin (PRL) signaling as a major pathogenic driver of adenomyosis. Specifically, we identified an epithelial cell subcluster, termed ECM-high epithelial cells, that was characterized by high extracellular matrix (ECM) gene expression and exhibited both epithelial and fibroblast dual characteristics. Notably, this ECM-high subcluster expanded with disease progression and showed exaggerated PRL signaling in adenomyosis patients. PRL treatment promoted the proliferation and inhibited the apoptosis of endometrial epithelial cells, which may facilitate lesion expansion. Moreover, analysis of fibroblasts revealed a proinflammatory subpopulation with augmented PRL signaling in patients. PRL treatment of endometrial fibroblasts increased inflammatory cytokine production, establishing the important role of hyperactivation of PRL signaling in the stromal inflammation associated with adenomyosis. The pathogenic effects of PRL signaling were further validated in transgenic mice overexpressing PRL and in mice with hyperactivation of PRL signaling induced by pituitary transplantation. In pituitary-isograft mice, we found that localized PRL signaling, rather than systemic PRL elevation, strongly influences adenomyosis development.

PRL signaling has been implicated in endometriosis, another condition characterized by the presence of ectopic endometrium.^18^ Thus, blockade of the PRL receptor (PRLR) has been shown to inhibit endometriosis in a mouse model and exhibits comparable efficacy to that of the antiestrogen drug Faslodex or the GnRH antagonist cetrorelix.^19^ In this study, we investigated the therapeutic potential of targeting the PRLR in adenomyosis by administering the monoclonal antibody HMI-115 to mice with intrauterine pituitary isograft-induced adenomyosis. This treatment significantly reduced the development and progression of adenomyosis. These findings underscore the potential of targeting PRL-PRLR signaling in the treatment of adenomyosis, paving a new avenue for therapeutic interventions.

## Results

### Identification of ECM-high epithelial cells in endometrial tissue

**Fig. 1:**
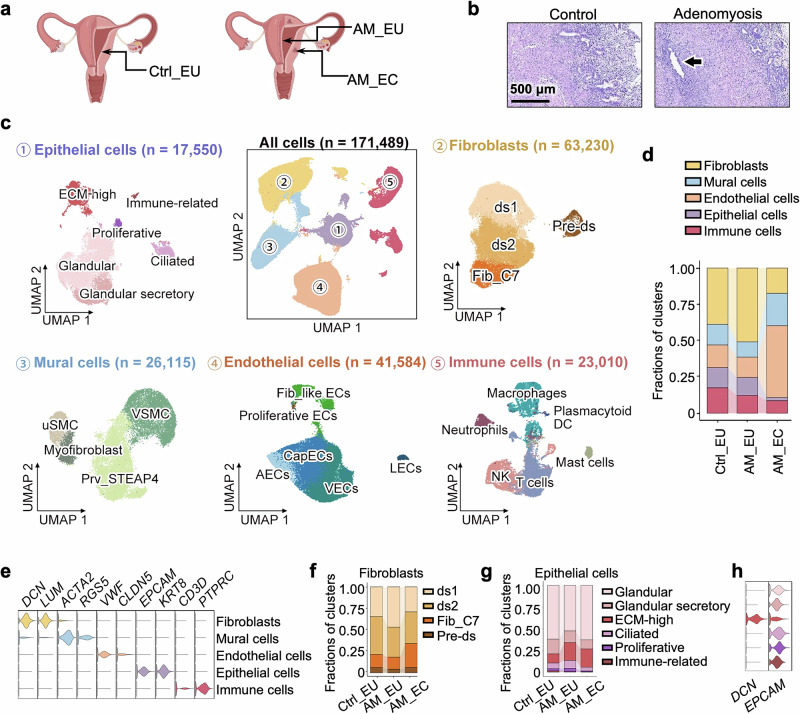
Identification of ECM-high epithelial cells in the endometrium. **a** Diagram showing the locations of uterine biopsies collected for scRNA-seq, including eutopic endometria from individuals without adenomyosis (Ctrl_EU) and eutopic (AM_EU) and ectopic (AM_EC) endometrial tissues from patients with adenomyosis. **b** Representative images of hematoxylin and eosin (H&E) staining of endometria from participants without (Control) or with (Adenomyotic) adenomyosis. The arrow indicates the ectopic endometrial gland. Scale bar, 500 μm. **c** Uniform manifold approximation and projection (UMAP) plot showing 171,489 single cells from eutopic and ectopic endometrial tissue samples (center UMAP plot), which were grouped into 5 cell types: epithelial cells, fibroblasts, mural cells, endothelial cells, and immune cells. Each cell type was further clustered into subpopulations, as shown in the radial UMAP plots. **d** Bar plot showing the fractions of major cell types among all the cells in different sample groups of Ctrl_EU, AM_EU, and AM_EC. **e** Violin plot showing the expression of the marker genes used for identifying the major cell types in this scRNA-seq dataset. **f** Bar plot showing the fractions of fibroblast subclusters in different sample groups. **g** Bar plot showing the fractions of epithelial subclusters in different sample groups. **h** Violin plot showing that ECM-high epithelial cells expressed both EPCAM and DCN

We analyzed uterine samples from 13 patients who underwent hysterectomy via scRNA-seq. To minimize the effects of menstrual cycle variability, all samples were collected during the secretory phase, which offered a comprehensive view of the cell composition of the endometrium. Control samples (Ctrl_EU) were obtained from the eutopic endometria of 7 individuals without adenomyosis, whereas samples from 6 adenomyosis patients included both eutopic (AM_EU) and ectopic (AM_EC) tissues (Fig. 1a). None of the participants had received hormonal medications for at least 3 months prior to surgery or sample collection (Supplementary Table 1).

Histological examination confirmed adenomyosis diagnosis by identifying endometrial gland invasion into the myometrium (Fig. 1b). Following quality control, a total of 171,489 cells were analyzed (Supplementary Fig. 1a, b) and annotated as epithelial cells, fibroblasts, mural cells, endothelial cells, or immune cells according to the corresponding marker genes (Fig. 1c–e). Although the cell type composition varied between eutopic and ectopic endometrial samples, consistency within each group was observed (Supplementary Fig. 1c).

The proportion of endothelial cells in the entire cell population was significantly increased in the AM_EC samples, indicating active angiogenesis in the lesions (Fig. 1d and Supplementary Fig. 1c). In particular, among the subclusters of endothelial cells, including capillary endothelial cells (CapECs), arterial endothelial cells (AECs), venous endothelial cells (VECs), lymphatic endothelial cells (LECs), proliferative endothelial cells (proliferative ECs), and endothelial cells expressing fibroblast marker genes (Fib_like ECs), CapECs exhibited the most significant increase in size in the lesions (Supplementary Fig. 2a, b). Another important component of the vasculature is mural cells. The mural cells were grouped into perivascular cells (Prv_STEAP4), vascular smooth muscle (VSMC), uterine smooth muscle (uSMC), and myofibroblasts (Fig. 1c and Supplementary Fig. 2c, d) according to previous studies.^20–22^ Crosstalk among mural cells and endothelial cells via VEGF signaling, especially between uSMCs/VSMCs and CapECs (Supplementary Fig. 2e), was markedly intensified in AM_ECs. This enhanced VEGF signaling may account for the increased angiogenic activity observed in the lesions and the abnormal bleeding associated with adenomyosis.

Previous studies of human endometrial stromal cells have identified a special subgroup of fibroblasts that express complement component 7 (*C7*). These Fib_C7 cells are enriched in the basal layer of the endometrium.^23^ Interestingly, the percentage of these Fib_C7s in fibroblasts was increased in the ectopic endometrial tissue of adenomyosis patients (Fig. 1f). In addition to Fib_C7, the remaining fibroblasts were further grouped into 3 subclusters: decidual stromal cell 1 (ds1), decidual stromal cell 2 (ds2), and predecidual stromal cells (Pre-ds). Subcluster Pre-ds were characterized by genes related to cell proliferation, such as *MKI67*, *TOP2A*, *CENPF*, *CDK1*, and *CCNB1* (Supplementary Fig. 2f). The ds1 cells were characterized by genes regulating cell proliferation, apoptosis, and migration, such as *MT2A*, *HMGA1*, *ETS2*, *GLIPR1*, and *CAV1*. The feature genes of the ds2 cells were those responsive to growth or stress stimuli (e.g., *EGR1*, *IER2*, *TXNIP*, *DNAJB1*, ZFP36, and *HSPA1B*). Moreover, the ds1 cells presented the highest expression of *DKK1* and *WNT4*, while the expression levels of steroid receptors were lower than those in the ds2 cells. Similarly, the expression of genes involved in both TGFβ and IGF signaling tended to decrease in ds1 cells compared with ds2 cells (Supplementary Fig. 2g), suggesting that ds1 cells are in a more differentiated state.^20^

In line with the pathological results, scRNA-seq confirmed the presence of epithelial cells in AM_EC samples (Fig. 1d and Supplementary Fig. 1c). To delineate the changes in epithelial cells in adenomyosis, we clustered epithelial cells into 6 subtypes: glandular, glandular secretory, ciliated, proliferative, immune-related, and ECM-high epithelial cells (Fig. 1g and Supplementary Fig. 3a). The most prominent difference in epithelial cells between adenomyosis patients and control subjects was an increase in the ECM-high population (Fig. 1g). These cells expressed both epithelial (*EPCAM*) and fibroblast (*DCN*) markers (Fig. 1h), and their signature biological processes and molecular functions were related to organizing or binding to the ECM (Supplementary Fig. 3b). Similar cells with epithelial and fibroblast dual signatures have also been identified by other studies (Supplementary Fig. 3c) and are present in the endometrium under physiological conditions.^21,24^

### Pathological alterations in ECM-high epithelial cells in adenomyosis

**Fig. 2:**
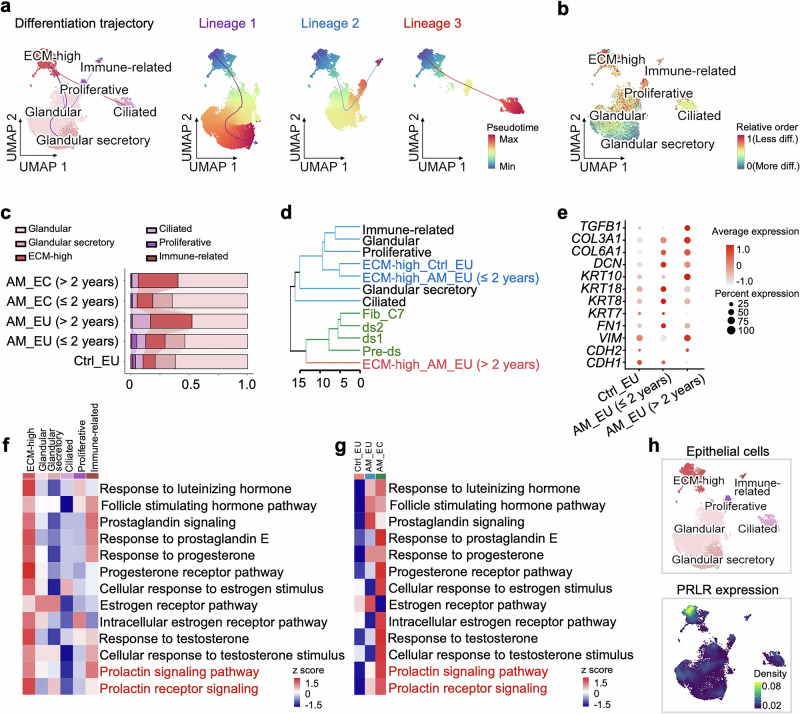
Pathological changes in ECM-high epithelial cells in adenomyosis. **a** Slingshot analysis showing the trajectory of the differentiation of epithelial cells with ECM-high epithelial cells as the shared starting cluster for 3 different lineages. **b** The relative development order of each epithelial subtype determined by CytoTRACE 2. **c** Bar plot showing the fractions of epithelial cell subclusters in the endometria of control individuals (Ctrl_EU) and eutopic (AM_EU) or ectopic (AM_EC) endometrial tissue of patients with a history of adenomyosis less than or equal to 2 years (≤ 2 years) or longer than 2 years (> 2 years). **d** Hierarchical clustering of epithelial and fibroblast subclusters. The ECM-high epithelial cells from adenomyosis patients (ECM-high_AM_EU) were further grouped according to the duration of the condition, with a history of less than or equal to 2 years designated ≤ 2 years and more than 2 years designated > 2 years. **e** Dot plot showing the *Z*-score-scaled mean expression of EMT-related genes, as well as epithelial and fibroblast marker genes, in ECM-high epithelial cells from the eutopic endometria of control (Ctrl_EU) and adenomyosis patients (AM_EU) with a disease history of less than or equal to 2 years or longer than 2 years. **f** Heatmap showing the results of gene set variation analysis (GSVA) of hormone-related pathways in subclusters of epithelial cells. **g** Heatmap showing the results of GSVA of hormone-related pathways in ECM-high epithelial cells from Ctrl_EU, AM_EU, and AM_EC. **h** Density plot showing the expression levels of PRLR in epithelial cells

The inference of the cell differentiation trajectory via Slingshot^25^ suggested that ECM-high cells have the potential to differentiate into diverse epithelial cell subtypes (Fig. 2a). Analysis with CytoTRACE 2^26^ also indicated that ECM-high cells exhibited higher differentiation potential relative to other epithelial subclusters (Fig. 2b). Notably, the increase in the proportion of ECM-high cells in the epithelial population was more pronounced in patients who had been diagnosed with adenomyosis for more than 2 years (Fig. 2c). The differentiation potential and expansion of the population with disease progression suggested that ECM-high epithelial cells may play an important role in the progression of adenomyosis.

Hierarchical clustering revealed that in the eutopic endometrium, the ECM-high epithelial cells of the control subjects and patients with a shorter disease duration (≤ 2 years) grouped with typical epithelial cells, whereas those from patients diagnosed with adenomyosis longer than 2 years (> 2 years) were clustered with fibroblasts (Fig. 2d), suggesting that these ECM-high cells gradually acquired more fibroblast-like characteristics as the disease progressed. Compared with those in Ctrl_EU, the repressed genes in AM_EU were consistently involved in epithelial morphogenesis and development, while processes related to the stress response and cell proliferation were activated (Supplementary Fig. 3d). Notably, the expression of marker genes of epithelial-to-mesenchymal transition (EMT) did not change consistently in the eutopic endometria of patients. Thus, *CDH1* was downregulated and *FN1* was upregulated, whereas the expression levels of *CDH2* and *VIM* were increased only in the AM_EU of patients with a longer disease history (Fig. 2e). Furthermore, the expression of major cytokeratins was not decreased in the patients’ ECM-high epithelial cells, although a panel of fibroblast feature genes (e.g., *DCN*, *COL3A1*, *COL6A1*, and *TGFB1*) were upregulated (Fig. 2e). These observations are in line with recent studies of endometriosis reporting few changes in the expression of EMT-related genes in the eutopic endometria of patients.^27,28^ Therefore, EMT may not be an early and causal event in adenomyosis or endometriosis.^27,29^

Adenomyosis is a well-known hormone-dependent condition, and gene set variation analysis (GSVA) indicated that ECM-high epithelial cells exhibited the strongest hormone responsiveness among all the epithelial subclusters (Fig. 2f). This cluster of cells in patients exhibited activation of several hormone signaling pathways in both eutopic and ectopic endometria, including those for luteinizing hormone, follicle-stimulating hormone, prostaglandin, estrogen, progesterone, and PRL (Fig. 2g). Notably, PRLR expression was highly enriched in ECM-high epithelial cells (Fig. 2h), whereas receptors of estrogen, progesterone, and androgen were uniformly expressed in all epithelial subclusters (Supplementary Fig. 3e), suggesting that high-ECM-related cells constitute the major epithelial subtype responsive to PRL signaling.

### Enhanced PRL signaling promotes the proliferation and survival of endometrial epithelial cells

**Fig. 3:**
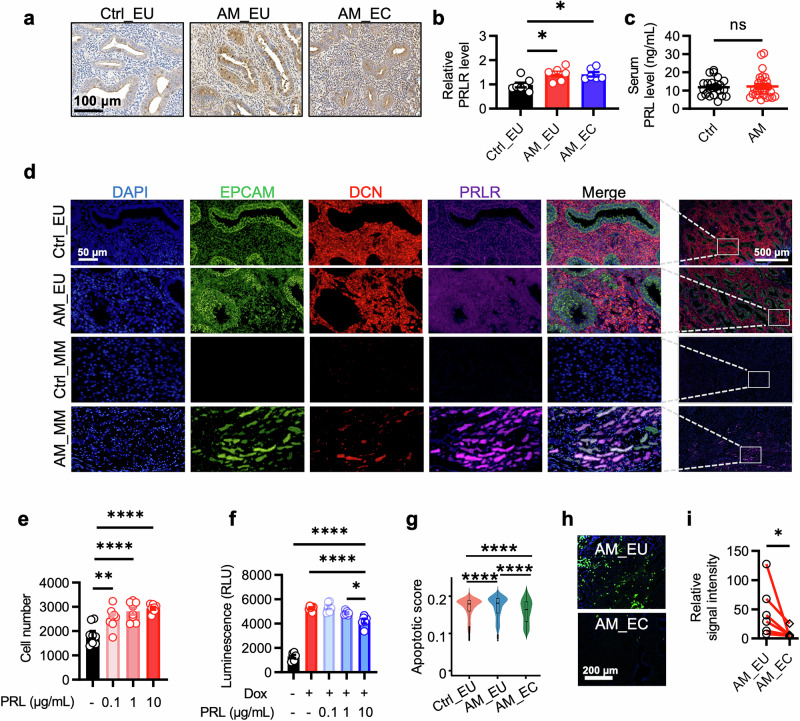
PRL signaling promotes proliferation and represses apoptosis of epithelial cells. **a**, **b** Representative images (**a**) and statistical results of the signal intensity (**b**) of the immunohistochemical staining of PRLR in human uterine sections. Scale bar, 100 μm. (Ctrl_EU, *n* = 7; AM_EU, *n* = 6; AM_EC, *n* = 6). **c** PRL levels in the sera of individuals with (AM) or without (Ctrl) adenomyosis. (Ctrl, *n* = 23; AM, *n* = 27) **d** Representative images of RNAscope results showing the expression of EPCAM, DCN, and PRLR in the eutopic endometrium (Ctrl_EU) and myometrium (Ctrl_MM) of control individuals as well as in the eutopic endometrium (AM_EU) and lesions in the myometrium (AM_MM) of adenomyosis patients. Scale bars, 50 μm in the panels of columns 1–5 and 500 μm in the last column. **e** Numbers of primary human endometrial epithelial cells treated with PRL at the indicated concentrations. (*n* = 8 for each group). **f** Statistical results of the luminescence assay measuring caspase-3 and -7 activities in primary human endometrial epithelial cells treated with doxorubicin in the presence or absence of PRL at the indicated concentrations. (*n* = 6 for each group). **g** Box and violin plots displaying the apoptosis scores of epithelial cells in the Ctrl_EU, AM_EU, and AM_EC groups. The upper and lower quartiles are indicated, with horizontal lines representing the median. The whiskers extend to the range of data within 1.5 times the interquartile range above the third quartile and below the first quartile. The sample sizes were as follows: Ctrl_EU: *n* = 9618; AM_EU: *n* = 6908; and AM_EC: *n* = 1024. **h**, **i** Representative images (**h**) and statistical results of the signal intensity (**i**) of TUNEL staining of the eutopic endometrium (AM_EU) and the corresponding lesions in the myometrium (AM_EC) from the same patient. Scale bar, 200 μm. (*n* = 6 for each group) All the data are presented as the means ± s.e.m.s. *P*-values were determined via one-way ANOVA with Tukey’s multiple comparison (**b**, **e**, and **f**), a two-sided unpaired *t*-test (**c**), a Kruskal‒Wallis test followed by Dunn’s test with Bonferroni correction (**g**), or a two-tailed paired Wilcoxon matched-pair test (**i**). *P < 0.05; **P < 0.01; ****P < 0.0001 compared with the corresponding controls

Immunohistochemical staining revealed increased expression of PRLR in the absence of increased serum PRL levels in adenomyosis patients (Fig. 3a–c and Supplementary Table 1). Furthermore, RNAscope staining confirmed the elevated PRLR expression and the presence of cells expressing both *EPCAM* and *DCN* (representing the ECM-high epithelial cells in the present study) in the myometrium, even in the absence of gland structure (Fig. 3d). These findings suggest that the appearance of ECM-high cells in the myometrium is an early event in adenomyosis development.

To determine the direct effects of increased PRL signaling, human primary endometrial epithelial cells with endogenous PRLR expression were incubated with PRL, which stimulated cell proliferation in a dose-dependent manner (Fig. 3e). Additionally, PRL treatment protected endometrial epithelial cells against doxorubicin-induced apoptosis (Fig. 3f). Consistent with these findings, the scRNA-seq data revealed that enhanced PRL signaling in ectopic lesions was associated with a reduced apoptotic score (Fig. 3g), which was further supported by the decreased TUNEL staining intensity in the lesions compared with the corresponding eutopic endometria of the same patient (Fig. 3h, i). Thus, the increased proliferation and repressed apoptosis of PRLR-expressing epithelial cells may collectively drive the expansion of ectopic endometrial lesions.

### Role of fibroblasts in promoting uterine inflammation, fibrosis, and structural disruption in adenomyosis

**Fig. 4:**
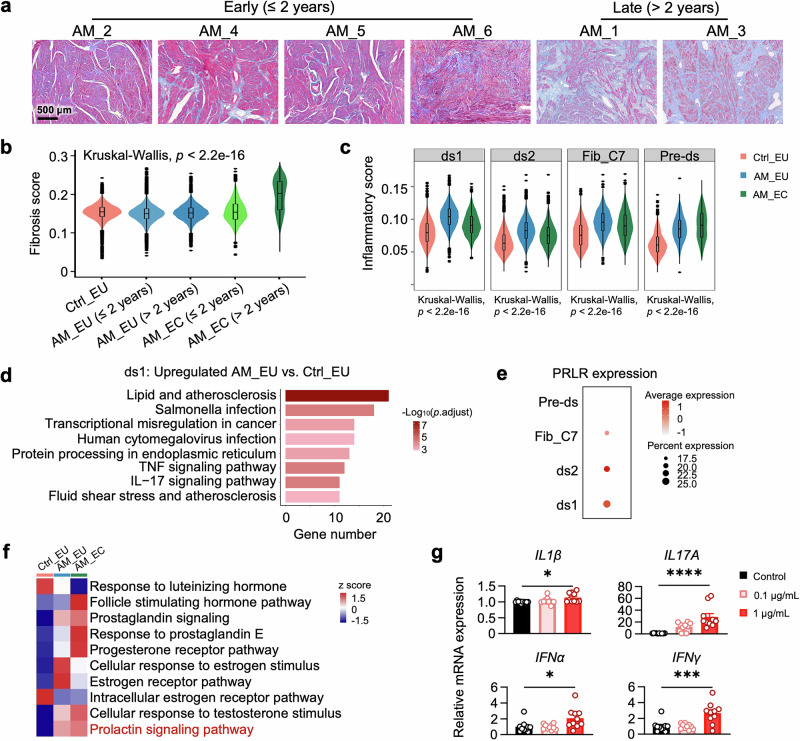
The fibroblast subcluster ds1 was enriched in the expression of inflammatory genes and augmented PRL signaling in adenomyosis. **a** Representative images of Masson’s trichrome staining of uterine sections from patients diagnosed with adenomyosis for less than or equal to 2 years (≤ 2 years) or more than 2 years (> 2 years). Scale bar, 500 μm. **b** Box and violin plots displaying the fibrosis scores of fibroblasts in the Ctrl_EU, AM_EU (≤ 2 years), AM_EU (> 2 years), AM_EC (≤ 2 years), and AM_EC (> 2 years) groups. The sample sizes were as follows: Ctrl_EU: *n* = 26,812; AM_EU (≤ 2 years): *n* = 19,362; AM_EU (> 2 years): *n* = 8922; AM_EC (≤ 2 years): *n* = 6744; and AM_EC (> 2 years): *n* = 1390. **c** Box and violin plots displaying the inflammatory scores across different fibroblast clusters. The sample sizes were as follows: Ctrl_EU (ds1: *n* = 9289; ds2: *n* = 11,800; Fib_C7: *n* = 4135; Preds: *n* = 1588), AM_EU (ds1: *n* = 13,260; ds2: *n* = 9875; Fib_C7: *n* = 4003; Pre-ds: *n* = 1146), and AM_EC (ds1: *n* = 2375; ds2: *n* = 2994; Fib_C7: *n* = 2265; Preds: *n* = 500). In the box plots of (**b**) and (**c**), the upper and lower quartiles are indicated, with horizontal lines representing the median. The whiskers extend to the range of data within 1.5 times the interquartile range above the third quartile and below the first quartile. **d** Bar plot showing the activated pathways enriched by Kyoto Encyclopedia of Genes and Genomes (KEGG) analysis of the upregulated genes in the ds1 cells of eutopic endometria from adenomyosis patients (AM_EU) compared with those from control subjects (Ctrl_EU). **e** Dot plot showing the *Z*-score-scaled mean expression of PRLR in each fibroblast subcluster. **f** Heatmap showing the results of GSVA of hormone-related pathways in ds1 cells. **g** Relative mRNA levels of inflammatory cytokines in hEM15A cells treated with vehicle (Control) or PRL at the indicated concentrations, as determined by RT‒qPCR. (*n* = 10 for each group). Statistical differences were assessed via the Kruskal‒Wallis test followed by Dunn’s test with Bonferroni correction in (**b**, **c**) or one-way ANOVA with Dunnett’s multiple comparisons test in (**g**). *P < 0.05; ***P < 0.001; ****P < 0.0001 compared with the corresponding controls

Another important pathological feature of adenomyosis is fibrosis of the uterine wall, which may compromise uterine elasticity and contribute to infertility and dysmenorrhea.^30^ Pathological examinations revealed worsening fibrosis in the uteri of patients diagnosed with adenomyosis for more than 2 years (Fig. 4a). Fibroblasts are recognized as the primary contributors to tissue fibrosis.^31^ Indeed, fibroblasts from patients with a longer history of adenomyosis presented elevated fibrosis scores, particularly those from patients with a longer history of adenomyosis (Fig. 4b). Among the fibroblasts, Fib_C7 had the highest score for fibrosis (Supplementary Fig. 4a). A previous study revealed that Fib_C7 cells are the predominant fibroblasts in endometrial ectopic ovarian lesions.^21^ Similarly, the proportion of Fib_C7 was increased in AM_ECs (Fig. 1f), which may contribute to aggravated fibrosis in adenomyosis. Furthermore, Fib_C7 is a specialized fibroblast subcluster enriched in the dense basal layer separating the functional layer from the myometrium^20,21,23^ and is crucial for maintaining the structural and functional integrity of the basal layer by producing ECM components. Intriguingly, the fibrosis score of Fib_C7 cells from patients’ eutopic endometria was moderately lower than that of control cells (Supplementary Fig. 4b), which was associated with repressed ECM and cell adhesion-related pathways enriched by Gene Ontology (GO) analysis (Supplementary Fig. 4c). This disruption in the ECM may disturb the integrity of the endometrial basal layer, thereby facilitating the invasion of endometrial components into the myometrium in adenomyosis patients.

Studies suggest that enhanced fibrosis might be a sequela of the inflammatory response.^32^ Thus, we evaluated the inflammatory gene set in all the major cell types. Interestingly, among all major cell types, fibroblasts presented the most significant increase in the inflammatory score, whereas immune cells presented only moderate or negligible changes (Supplementary Fig. 4d), indicating that fibroblasts are a major source of the augmented inflammation associated with adenomyosis. In particular, subcluster ds1 fibroblasts presented the highest inflammatory score under both basal conditions and adenomyosis (Fig. 4c). Consistently, differential gene expression analysis highlighted several inflammation-related genes as the top feature genes of ds1 (Supplementary Fig. 4e) and inflammatory pathways as the major activated processes in AM_EU relative to Ctrl_EU in the ds1 population (Fig. 4d), corroborating the involvement of ds1 cells in adenomyosis-related inflammation. Remarkably, the ds1 cells presented increased expression of PRLR and increased PRL signaling in patients (Fig. 4e, f). In addition, treating human endometrial stromal hEM15A cells with PRL induced the expression of a group of inflammatory cytokines associated with adenomyosis (Fig. 4g),^33^ suggesting that PRL signaling contributes to enhanced inflammatory responses in fibroblasts.

In addition to fibrosis and inflammation, the physiological functions of fibroblasts are disrupted in adenomyosis. Importantly, the communication between fibroblasts and epithelial cells was impaired. First, WNT and NOTCH signaling mediated by fibroblasts regulates epithelial cell fate during the menstrual cycle regeneration.^23^ In adenomyosis patients, both WNT and NOTCH signaling were markedly abrogated (Supplementary Fig. 5a, b). Furthermore, communication between fibroblasts and epithelial cells via cell adhesion signaling, such as junctional adhesion molecule (JAM) and cadherin (CDH), was downregulated in adenomyosis patients (Supplementary Fig. 5c-d). Repressed JAM signaling has been implicated in the proliferation and migration of keratinocytes^34^ and cancer cells^35^ and represses intercellular interactions via E-cadherin, directly impairing cell attachment and facilitating migration.^36^ These results suggest that the proper interaction of endometrial epithelial cells with their surrounding environment is disrupted.

Taken together, these findings demonstrated that fibroblasts in adenomyosis undergo profound changes, becoming more inflammatory and fibrogenic while losing their physiological functions in regulating epithelial cell adhesion, migration, and differentiation. In particular, PRL stimulated the production of inflammatory cytokines in cultured endometrial fibroblasts. Moreover, PRLR-enriched ds1 fibroblasts presented the most significant increase in the inflammatory score in patients, which was associated with hyperactivation of PRL signaling. These findings suggest that PRL signaling may play a role in the fibroblast-mediated pathological manifestations of adenomyosis.

### Enhanced prolactin signaling drives adenomyosis development

**Fig. 5:**
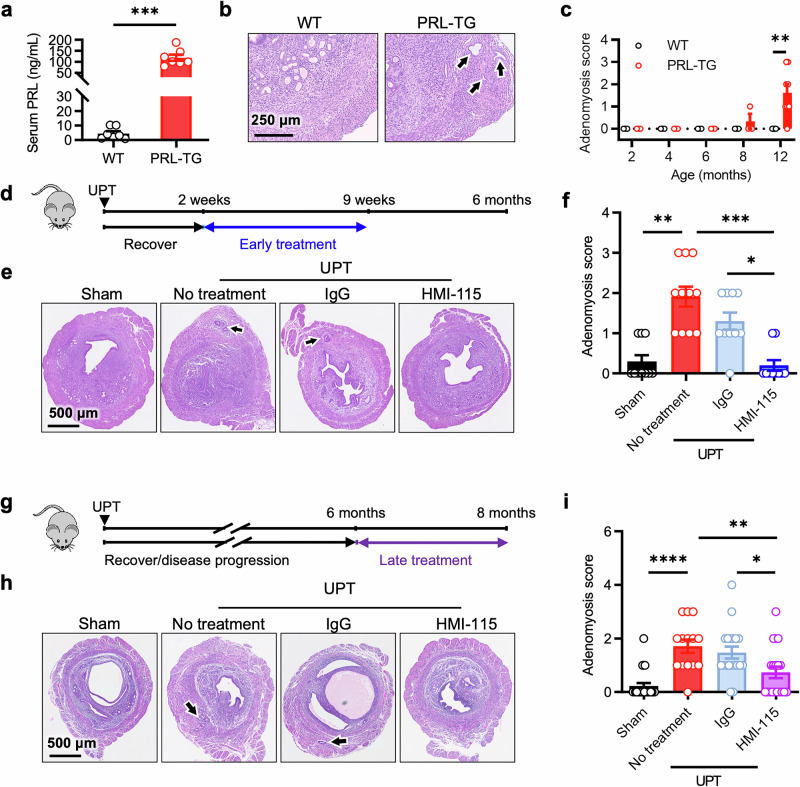
High levels of PRL trigger, while blocking the PRLR alleviates adenomyosis in animal models. **a** Serum PRL levels in PRL transgenic (PRL-TG) mice and their wild-type (WT) littermates. (*n* = 7 for each group). **b** Representative images of H&E-stained uteri from WT and PRL-TG mice. The arrows indicate the ectopic endometrial glands. Scale bar, 250 μm. **c** Adenomyosis scores of mice at the indicated ages. (2–8 months, *n* = 3 for each genotype; 12 months, WT = 5; PRL-TG, *n* = 7). **d**, **f** Experimental design of early treatment (**d**), representative images of H&E-stained uteri (**e**), and adenomyosis scores (**f**) of mice subjected to sham surgery (Sham) or uterine horn pituitary transplantation (UPT) and treated with saline (No treatment), IgG, or HMI-115. The arrows indicate the ectopic endometrial glands. Scale bar, 500 μm. (Sham, *n* = 10; No treatment, *n* = 11; IgG or HMI-115, *n* = 10). **g–i** Experimental design of late treatment (**g**), representative images of H&E-stained uteri (**h**), and adenomyosis scores (**i**) of mice subjected to sham surgery (Sham) or UPT and treated with saline (No treatment), IgG, or HMI-115. The arrows indicate the ectopic endometrial glands. Scale bar, 500 μm. (Sham, *n* = 22; No treatment, *n* = 14; IgG, *n* = 19; HMI-115, *n* = 19). All the data are presented as the means ± s.e.m.s. *P*-values were determined via two-tailed unpaired *t*-tests (**a**), multiple Mann‒Whitney tests (**c**), or one-way ANOVA with Dunn’s multiple comparisons (**f**, **i**). *P < 0.05; **P < 0.01; ***P < 0.001; ****P < 0.0001 compared with the corresponding controls

To determine whether enhanced PRL signaling alone could induce adenomyosis, we generated PRL transgenic (PRL-TG) mice with increased circulating PRL levels (Fig. 5a) and evaluated their uteri at various time points via a grading system^37^ to measure the degree of myometrial invasion by endometrial tissue (Supplementary Fig. 6a). Over 80% of the PRL-TG mice had developed adenomyosis by 12 months of age, whereas their wild-type (WT) littermates exhibited normal uterine structures, indicating that enhanced PRL signaling is sufficient to trigger adenomyosis development (Fig. 5b, c). We further utilized a mouse model in which the pituitary gland, the primary source of circulating PRL, was transplanted either under the renal capsule or into the uterine horn (Supplementary Fig. 6b). In the renal capsule pituitary transplant (RPT) model, the transplanted pituitary gland releases hormones into the bloodstream, increasing circulating PRL levels; conversely, in the uterine horn pituitary transplant (UPT) model, PRL effects are localized without significantly altering serum PRL levels (Supplementary Fig. 6c). Histological assessment of the uteri at 2, 4, or 6 months postsurgery revealed progressive adenomyosis development. By 6 months, all UPT model mice had developed adenomyosis, whereas only a minority of RPT model mice had pathological changes, underscoring the critical role of localized PRL signaling in adenomyosis pathogenesis (Supplementary Fig. 6d).

Given the TIAR hypothesis, which links uterine injury to adenomyosis development,^38^ we explored whether surgical trauma contributes to the pathogenesis of adenomyosis. Uterine horns that underwent surgery without pituitary transplantation developed adenomyosis less frequently than those that received a transplant (Supplementary Fig. 6e), highlighting that exacerbated local PRL signaling, rather than mechanical injury alone, triggers the development of the disease.

### Blocking PRL signaling alleviates adenomyosis

To rule out the involvement of other pituitary-derived factors in the pathogenesis of adenomyosis, we used HMI-115, a monoclonal antibody that targets PRLR, to specifically block PRLR-mediated signaling. Moreover, we explored the therapeutic potential of targeting the PRLR for adenomyosis treatment. HMI-115 was administered 2 weeks post-UPT surgery, before any pathological changes were evident. This early intervention significantly reduced the development and severity of adenomyosis compared with those of controls treated with human IgG (Fig. 5d–f).

In more therapeutic settings, HMI-115 was administered 6 months postsurgery, when adenomyosis had already been established (Fig. 5g and Supplementary Fig. 6d). Even with this late treatment, HMI-115 effectively reduced disease severity, with approximately half of the treated mice showing no pathological signs of ectopic lesions (Fig. 5h, i). In addition, late treatment with HMI-115 attenuated the increase in PRLR expression induced by pituitary transplantation (Supplementary Fig. 6f). Importantly, neither early nor late treatment affected body or uterine weight (Supplementary Fig. 6g, h).

Furthermore, we evaluated the levels of IL-4^39^ and CA-125,^40^ which have been shown to be elevated in the uteri of adenomyosis patients. In the UPT model, IL-4 levels were elevated in the uteri of UPT mice and were reduced following late HMI-115 treatment (Supplementary Fig. 6i). However, CA-125 was not significantly altered after pituitary transplantation and was not affected by HMI-115 (Supplementary Fig. 6j).

## Discussion

Despite the high prevalence of adenomyosis, our understanding of this painful gynecological disorder is still limited to hypotheses lacking solid evidence. Using scRNA-seq, we examined the various cell types and subtypes involved in adenomyosis and revealed their contributions to symptoms. Notably, adenomyosis and control endometrial tissue samples were collected via hysterectomy—an irreversible procedure with profound implications in women. Therefore, the sample size of this study was modest. However, with integrated approaches, we identified PRL signaling as a key pathological driver and pinpointed the specific cell types affected by its dysregulation. In particular, we characterized a subcluster of epithelial cells with high ECM, enriched PRLR expression, and an expanded population in adenomyosis patients. The increase in the ECM-high epithelial population within lesions appeared to be driven by exaggerated PRL signaling, which enhanced the proliferative and antiapoptotic capabilities of these epithelial cells. Importantly, we discovered a highly inflammatory subcluster of fibroblasts, ds1. Similar to ECM-high cells, ds1 fibroblasts exhibit high levels of PRLR expression with augmented PRL signaling in patients. Furthermore, cytokine production was stimulated by PRL in endometrial fibroblasts with PRLR expression. Using animal models, we demonstrated that increased PRL signaling was sufficient to induce adenomyosis, while blocking the PRLR markedly ameliorated adenomyosis manifestations. These findings indicate that PRL-PRLR signaling is an important therapeutic target for the treatment of adenomyosis (Supplementary Fig. 6k).

### Pathogenic significance of enhanced PRL-PRLR signaling

Although PRL signaling has been implicated in uterine abnormalities, including adenomyosis,^41–44^, the direct causal role of exaggerated PRL signaling in the pathogenesis and development of adenomyosis has not been established. A few pilot studies have explored the use of bromocriptine, a dopamine agonist, to treat adenomyosis by suppressing PRL production.^45–47^ However, activating dopamine signaling may alleviate adenomyosis symptoms via mechanisms other than attenuating PRL signaling.^30^ Moreover, in addition to adverse effects, some patients are resistant to bromocriptine treatment,^48,49^ limiting its therapeutic potential and suggesting that the source of PRL may be a critical factor. In this study, we have shown, for the first time, that increased PRL is sufficient to trigger adenomyosis in PRL-TG mice. Furthermore, we demonstrated that localized PRL excess was more potent than systemic PRL elevation in driving adenomyosis. In fact, we did not observe elevated serum PRL levels in adenomyosis patients. In this context, the number of PRLR-positive cells and the level of PRLR expression might play significant roles in disease progression. In line with this notion, we and others have observed increased expression of PRLR in patients and animal models of adenomyosis.^42,50^ More importantly, blocking PRLR with a specific monoclonal antibody markedly ameliorated the severity of pituitary transplant-induced adenomyosis. These findings define a causal role of PRL signaling in the pathogenesis of adenomyosis.

Mechanistically, we highlighted 2 major subtypes of cells with high levels of PRLR expression; thus, these cells are the most responsive to PRL signaling. These insights are crucial for developing targeted therapies for adenomyosis. The first group consists of ECM-high epithelial cells that display both epithelial and fibroblast characteristics. Cells with dual signatures have also been identified in a mouse model mimicking menstruation,^24^ and similar EPCAM- and DCN-expressing cells are present in normal human endometrial tissue,^21^ indicating that cells with dual characteristics do exist in the normal endometrium. Under pathological conditions, however, especially in patients with a longer history of adenomyosis, ECM-high epithelial cells acquire a more fibroblast-like transcriptome. Interestingly, Slingshot and CytoTRACE 2 both suggested that ECM-high epithelial cells presented the greatest differentiation plasticity among the epithelial subclusters. These results indicate the broad differentiation potential of ECM-high epithelial cells but do not support the interpretation of the high level of ECM as common progenitors for all epithelial lineages without further evidence.

While we cannot exclude the possibility that ECM-high epithelial cells originate from residual embryonic Müllerian ducts or adult stem cells, our data support the hypothesis that these cells are derived from the invagination of the endometrium into the myometrium. This finding is supported by the observation that WNT and NOTCH signaling are severely impaired, leading to disrupted control of epithelial cell differentiation and migration during each menstrual cycle. In addition, the production of ECM by Fib_C7, the major subcluster in the basalis layer, is abrogated, creating a less rigid ECM structure at the junction that may facilitate endometrial cell invasion into the myometrium. Importantly, regardless of the initial trigger, we have shown that PRL signaling drives hyperproliferation, attenuates apoptosis, and diminishes cell adhesion in epithelial cells. These changes collectively promote lesion expansion.

The second PRLR-enriched cell group was the fibroblast ds1. The ds1 cells demonstrated prominent upregulation of inflammation-related genes in adenomyosis patients. Thus, several inflammation-associated pathways were among the top activated processes in the ds1 of AM_EU compared with Ctrl_EU. More importantly, PRL induced the expression of several cytokines in cultured endometrial stromal cells with endogenous PRLR expression. Notably, previous studies have demonstrated that PRL promotes the proliferation of smooth muscle cells and the invasion of stromal cells in vitro.^51,52^ Therefore, PRL signaling also plays a role in the pathological changes in fibroblasts in adenomyosis.

Taken together, overactivated PRL-PRLR signaling constitutes a major mechanism underlying the development and progression of adenomyosis.

### Therapeutic potential of PRLR blockade

Adenomyosis is a hormone-dependent disorder, and current mainstream treatments involve medications that modulate estrogen or progesterone signaling. However, these treatments often have serious adverse effects due to the suppression of ovarian function^53^ and are particularly unsuitable for patients who wish to preserve fertility. Given the diurnal oscillation of PRL^54^, targeting PRLR could be a more effective therapeutic approach. Indeed, the administration of HMI-115, a monoclonal antibody against PRLR, significantly curtailed the progression of adenomyosis in our mouse models, lowering both the incidence and severity of the condition. The therapeutic efficacy of HMI-115 in an animal model suggests that blocking PRLR could be a viable strategy for managing adenomyosis in humans. Notably, preclinical tests^19^ and phase I clinical trials of HMI-115 (named BAY 1158061 in the reference)^55^ have identified no safety concerns. Furthermore, in people with genetic or acquired (due to pituitary lesions) PRL deficiency, other than the absence of lactation after delivery, no other clinical complaint has been reported.^56^ Collectively, these results suggest that the off-target effects of HMI-115 treatment are negligible, although PRLR is widely expressed in multiple tissue types.

In summary, we identified PRL-PRLR signaling as a major pathological driver of adenomyosis. By dissecting different cell types in the endometrium, we revealed the cellular and molecular mechanisms underlying the action of PRL. We further demonstrated the pathogenic effects of enhanced PRL-PRLR signaling in animal models and conducted a proof-of-concept study using an antibody targeting PRLR for the treatment of adenomyosis. These findings illuminate the cellular dynamics of adenomyosis and pave the way for new treatments that could significantly improve outcomes for patients suffering from this debilitating condition.

## Materials and methods

### Human subjects

This study was approved by the Ethics Committees of Peking Union Medical College Hospital (JS-1698) and Dazhou Central Hospital (2024189), and was conducted in accordance with all ethical guidelines involving human participants. All participants provided written informed consent and agreed to the unrestricted public sharing of their information in public databases.

The inclusion criteria for patients with uterine adenomyosis were as follows: (i) regular menstrual cycles, with sampling occurring during the secretory phase; (ii) absence of endometriosis, uterine cancer, uterine fibroids, and polycystic ovary syndrome; (iii) no hormone therapy within 3 months prior to surgery; (iv) normal karyotype and no viral infections; and (v) no use of intrauterine devices or hormonal contraceptives within the last 3 months. The control group was subjected to the same inclusion criteria. The participants in the control group underwent surgery due to cervical intraepithelial neoplasia. Postoperative histological analysis of the uterine samples confirmed the absence of cancer, adenomyoma, endometriosis, or adenomyosis.

Tissue samples were obtained from patients who underwent hysterectomy during the secretory phase of the menstrual cycle. Patients in the luteal phase were first selected on the basis of their preoperative serum progesterone levels. The cycle phase was further confirmed via histological examination of paraffin-embedded sections of the endometrium after surgery. Adenomyosis in all patients was confirmed through pathological examination of the resected tissues. Freshly excised tissues were immediately preserved in MACS Tissue Storage Solution (Miltenyi, 130-100-008) and kept on ice until further processing. The information of all the participants is detailed in Supplementary Table 1.

### Animal models

All animal experiments were performed in accordance with the protocol approved by the Institutional Animal Care and Use Committee of Peking University (Protocol# FT-XiaoRP-15) and conformed to the Guide for the Care and Use of Laboratory Animals (NIH publication Np. 86-23, revised 1985). The mice had free access to a standard chow diet and water and were maintained under a 12-h light/dark cycle at 23 ± 2°C with 40–60% humidity in a barrier facility at the Laboratory Animal Center of Peking University, Beijing, China (an AAALAC-accredited experimental animal facility).

### PRL transgenic mice

PRL transgenic mice were generated via the use of a transgenic construct of full-length mouse PRL cDNA cloned and inserted into the pCAG expression vector. The linearized PRL expression vector was injected into fertilized C57BL/6N mouse embryos via microinjection. For genotyping, the sequences of the primers used were 5’-TCCTCAGTTTGGTGCAGTCC-3’ and 5’-GGGCTTCATGATGTCCCCAT-3’. Female PRL-TG mice and their wild-type littermates were examined at the indicated ages.

### Mouse model of adenomyosis induced by pituitary grafting

Two surgical models of pituitary transplantation were employed: pituitary transplantation under the renal capsule (RPT) and transplantation into the uterine horn (UPT). Specifically, 7-week-old female C57BL/6N mice weighing between 16 and 18 g were used as donors and recipients. The pituitary glands were harvested from anesthetized donor mice, rinsed gently in physiological saline, and set aside for transplantation. The recipient mice were anesthetized and positioned dorsal side up. For RPT, a small incision was made with surgical scissors to expose the kidney, followed by opening the renal capsule with a pair of fine-tipped forceps. The donor pituitary gland was inserted, and the capsule was subsequently sutured. For the UPT, a small incision was made with surgical scissors on the lower back near the ovarian region to expose the uterine horn. The pituitary gland was placed into the uterine horn via a puncture needle. The control side of the uterus underwent the same procedure and was punctured with a needle only, without a pituitary gland being transplanted.

The surgical wounds of the recipient mice were sutured, and the mice were placed on a heated pad to recover. Once they regained consciousness, they were transferred to a housing cage for postoperative care.

For treatment, 2 weeks (early treatment) or 6 months (late treatment) after UPT surgery, the mice were administered human IgG or HMI-115 at 30 mg/kg or the same volume of saline (untreated) once a week for 7 weeks.

### Scoring of adenomyosis in mouse models

The scoring system reflects the progression of adenomyosis and was determined as previously described.^37^ Specifically, no adenomyosis phenotype, 0 points; loss of concentricity in the inner myometrium, 1 point; endometrial stroma and glands infiltrating the inner myometrium, 2 points; endometrial stroma and glands infiltrating between the inner and outer myometrium, 3 points; endometrial stroma and glands infiltrating the outer myometrium, 4 points; and endometrial stroma and glands penetrating the outer myometrium and directly contacting the peritoneum, 5 points. One section from each segment was randomly selected for assessment, and the highest score of each section was used to calculate the average score. The average of 3 sections was used as the adenomyosis score of the corresponding mouse.

### Single-cell RNA sequencing and data analysis

#### Tissue dissociation

Fresh tissue samples were immediately processed for single-cell RNA sequencing (scRNA-seq). Tissues from patients with adenomyosis were collected from the eutopic endometrium (AM_EU) and lesions of the ectopic endometrium (AM_EC), whereas control samples were obtained from the eutopic endometria of participants without adenomyosis (Ctrl_EU). The preparation of single-cell suspensions for library construction was achieved through mechanical dissociation and enzymatic digestion. The detailed procedure was as follows: The tissue samples were minced into small pieces on ice and transferred into a centrifuge tube containing 10 mL of enzyme mixture (0.2% (w/v) collagenase and 0.05% DNase I dissolved in RPMI 1640 medium). The tissues were digested at 37 °C for 1.5 h. Following digestion, the dissociated cells were filtered through a 70 μm strainer prewetted with RPMI 1640 and collected in a 50 mL conical tube. The cells were resuspended by gentle pipetting and filtered again via a strainer. The tube walls were rinsed with additional RPMI 1640 to ensure the complete transfer of the cell suspension. The cell suspension was then centrifuged at 300 × *g* for 7 min at 4 °C, and the supernatant was discarded. The cell pellet was resuspended in 10 volumes of ice-cold 1× Red Blood Cell Lysis Solution and incubated on ice at 4 °C for 10 min to lyse any remaining erythrocytes. Subsequently, 10 mL of ice-cold PBS containing 0.04% BSA was added, and the cells were centrifuged at 300 × *g* for 10 min at 4 °C. The supernatant was discarded, and the cell pellet was gently resuspended in an appropriate volume of PBS containing 0.04% BSA for downstream applications.

### Single-cell capture, library preparation, and sequencing

The single-cell suspensions were analyzed for viability, aggregation rate, and cell count via a Countstar Biotech automated cell counter. Single-cell capture, barcoding, and library preparation were conducted via 10x Chromium v3.1 chemistry according to the manufacturer’s protocol. The resulting libraries were sequenced on an Illumina NovaSeq 6000 platform via an S2 100-cycle kit, aiming for a target of 50,000 reads per cell for the tissue samples.

### Data processing

The raw sequencing data were processed via Cell Ranger software (version 5.0.0, 10x Genomics) for demultiplexing, alignment to the GRCh38 human reference genome, and generation of unique molecular identifier (UMI) matrices. The outputs from Cell Ranger were subsequently imported into Seurat (version 4.0.4) via the ‘Read10X’ function.

Within each sample, cells with UMI counts in the upper 2% were excluded to eliminate potential outliers. Cells with fewer than 200 UMIs and those with a mitochondrial RNA content exceeding 20% were also filtered out on the basis of quality control criteria. After these filtering steps, a total of 171,489 cells from 13 subjects were selected for further analysis. Batch effects across samples were effectively mitigated via the Harmony method (version 0.1.0).

The preprocessed data underwent normalization and scaling with the Seurat function ‘NormalizeData’, followed by the identification of highly variable genes via ‘FindVariableFeatures’. Principal component analysis (PCA) was performed via ‘RunPCA’. Clustering was explored via the ‘FindClusters’ function with resolutions ranging from 0.1–1.2, with the optimal resolution determined on the basis of cluster stability assessed via the clustree (version 0.5.0) R package. This process resulted in the identification of 39 distinct clusters at a resolution of 1.2. Dimensionality reduction was further achieved via UMAP, as implemented by ‘RunUMAP’, to visualize the distribution of cells by placing those with similar local neighborhoods within dimensions 1-40.

Cell type annotation of major cell types and subclusters was conducted by leveraging the expression patterns of differentially expressed genes (Supplementary Tables 2–7) and aligning these with marker genes curated from the literature.

### Cell‒cell interactions

CellChat R package (version 1.5.0) was used to dissect the cell‒cell interaction networks among various cell types. For the subsequent analysis, we selectively identified receptors and ligands that were expressed in more than 10% of the cells within each specific cluster, ensuring a robust threshold for the investigation of significant intercellular communication pathways.

### Gene set scoring analysis

Gene sets associated with key cellular functions were retrieved from the Molecular Signatures Database (MsigDB, version 7.5, https://www.gsea-msigdb.org/gsea/msigdb/). The HALLMARK_INFLAMMATORY_RESPONSE gene set (v2024.1.Hs.gmt), GOCC_COLLAGEN_CONTAINING_EXTRACELLULAR_MATRIX gene set (v2024.1.Hs.gmt), and GOBP_APOPTOTIC_PROCESS (v2024.1.Hs.gmt) were used to score inflammation, fibrosis, and apoptosis, respectively. Gene activity for each set was calculated via the ‘AUCell_calcAUC’ function from the AUCell R package, which scores cells on the basis of the enrichment of genes within each gene set. Statistical differences were assessed via the Kruskal‒Wallis test.

### Gene set variation analysis (GSVA) and enrichment analysis

Pathway analyses were conducted with a focus on the hallmark pathways delineated within the Molecular Signatures Database (MsigDB, version 7.5, https://www.gsea-msigdb.org/gsea/msigdb/) leveraging the GSEABase software package (version 1.58.0) for data extraction. To ascertain pathway activities at the single-cell level, we employed GSVA, applying the standard parameters as defined within the GSVA software suite (version 1.22.4). Gene Ontology (GO) and Kyoto Encyclopedia of Genes and Genomes (KEGG) enrichment analyses were carried out via ‘enrichGO’ and ‘enrichKEGG’ with the R package clusterProfiler (version 4.6.2).

### Hierarchical clustering

Hierarchical clustering was performed on both epithelial and fibroblast subclusters. Among the epithelial cells, only the ECM-high epithelial cells were further subdivided into disease history ≤ 2 years or >2 years for more detailed analysis. Other epithelial subclusters, as well as all fibroblast subclusters, were not stratified by disease stage. Clustering was conducted via the ‘hclust’ function in R (version 4.2.2) on the basis of the top 2000 highly variable genes across the subclusters. The results were visualized via the Factoextra package (version 1.0.7).

### Slingshot

Trajectory analysis was conducted via the Slingshot R package (version 2.4.0)^25^ with default parameters. For this purpose, clustering information obtained from Seurat objects was directly input into Slingshot’s main function.

### CytoTRACE 2

To assess the cellular potency categories and developmental potential, we performed CytoTRACE 2 (version 1.1.0) analysis,^26^ an interpretable deep learning method for predicting the differentiation state from scRNA-seq data, with default parameters.

### Histology and immunohistochemical staining

Human tissue samples were obtained from Peking Union Medical College Hospital. Formalin-fixed paraffin-embedded (FFPE) tissues were sectioned into 6 μm slices, mounted on slides, and stained with hematoxylin and eosin (H&E) or Masson’s trichrome. The slides were subsequently scanned via a Carl Zeiss slide scanner at ×20 magnification for histopathological analysis.

Immunohistochemical staining was conducted on the FFPE tissue sections. Prior to staining, the sections were deparaffinized in xylene and rehydrated sequentially in 100%, 95%, and 70% ethanol. This was followed by microwave antigen retrieval, inactivation of endogenous peroxidase, and blocking of nonspecific binding sites. For immunohistochemistry (IHC) staining, the sections were incubated with PRLR primary antibodies overnight at 4 °C, followed by incubation with horseradish peroxidase (HRP)-conjugated secondary antibodies and development with 3,3’-diaminobenzidine (DAB) substrate after washing.

For TUNEL staining, after deparaffinization and rehydration, the tissue sections were treated with proteinase K at room temperature for 15 min. The sections were then incubated with 50 μL of TUNEL reaction mixture at 37 °C for 60 min in the dark. Following mounting with anti-fade mounting medium, the slides were scanned and analyzed with ImageJ.

### RNAscope

RNAscope was utilized for the detection of target gene mRNAs within cells via in situ hybridization. This technique was performed via the ACD Bio 3-plex RNAscope Kit (Cat No: 323100) following the manufacturer’s protocol. Briefly, tissue sections were pretreated, including baking, deparaffinization, and target retrieval, to increase mRNA accessibility and preserve tissue morphology. The sections were then hybridized with a mixture of target-specific oligonucleotide probes designed to hybridize to the mRNA of interest. This was followed by a series of amplification steps, including hybridization of preamplifier and amplifier molecules, leading to the binding of horseradish peroxidase (HRP)-labeled probes. Signal amplification was achieved via sequential amplification and HRP reactions, which allowed for the robust detection of low-abundance transcripts. Finally, the slides were counterstained with DAPI to visualize the cell nuclei and mounted with DAPI-containing anti-fade mounting medium. The fluorescent signals were subsequently analyzed via fluorescence microscopy.

### Cell proliferation and apoptosis

For proliferation and apoptosis assays, human primary endometrial epithelial cells (CPH-058) were purchased from Procell and cultured in specialized medium (CM-H058). The culture vessels were coated with mouse tail collagen type I. Cells were seeded at a density of 2000 cells per well in a 96-well plate. After attachment, the cells were treated with PRL. Following a 48-h incubation, live-cell counting was performed via high-content live-cell imaging.

For the apoptosis assay, Caspase-3/7 signaling was assessed via the Promega G8091 assay kit. After equilibrating the reagents in the G8091 kit to room temperature, a 100 μL mixture of solutions A and B was added to each well of the 96-well plate. The plate was gently shaken for 30 s and incubated at room temperature for 1 h. After incubation, the supernatant was transferred to a white opaque 96-well plate, and luminescence was measured at 490 nm.

### RNA isolation and RT‒qPCR analysis

Endometrial stromal cells (hEM15A) provided by Dr. Xiaohong Chang (People’s Hospital of Peking University, Beijing, China) were cultured in DMEM/F12 (Gibco, Cat No: C11330500BT) supplemented with 15% FBS (Vivacell, Cat No: C04001-500) and penicillin‒streptomycin (Beyotime, Cat No: C0222) and maintained at 37 °C with 5% CO_2_ in a humidified incubator. The cells were seeded in 24-well plates at a density of 40,000 cells per well. After attachment, the cells were treated with PBS or PRL at 0.1 μg/mL or 1 μg/mL for 24 h. The cells were then harvested, and total RNA was extracted via an NcmSpin Cell/Tissue Total RNA Kit (NCMbiotech, Cat No: M5105). The RNA was reverse-transcribed with random primers via HiScript IV All-in-One Ultra RT SuperMix, and quantitative PCR (qPCR) was performed via ChamQ SYBR qPCR Master Mix (Vazyme, Cat No: Q321-02) on a Roche LightCycler 96. Relative mRNA expression levels were determined via the 2^−ΔΔCT^ method, with normalization to GAPDH expression. The sequences of primers used for qPCR are listed in Supplementary Table 8.

### Statistical analyses

Statistical analyses were performed via R (version 4.0.5) or GraphPad Prism (version 9.0.1). The data are presented as the means ± s.e.m.s. Statistical significance was determined via the Kruskal‒Wallis test, Wilcoxon test, one-way ANOVA with Tukey’s multiple comparison test, one-way ANOVA with Dunnett’s multiple comparison test, or *t*-test, as specified in the figure legends.

## Supplementary information

PRLR in adenomyosis_Supplementary information

## Data Availability

The scRNA-seq data have been deposited in GSA-Human (http://ngdc.cncb.ac.cn/gsa-human) under the accession number HRA008245. All other data supporting the findings of this study are available from the corresponding authors upon reasonable request.
