# Supplementary material for: Single-cell RNA sequencing identifies the prolactin receptor as a therapeutic target in adenomyosis
Source: Signal Transduct Target Ther. 2025 Aug 13;10:258. doi: 10.1038/s41392-025-02339-z (PMC12350698; doi:10.1038/s41392-025-02339-z)
Supplement: Supplementary file 1 — PRLR in adenomyosis_Supplementary information [file 41392_2025_2339_MOESM1_ESM.docx]

Supplementary Materials for

**Single-cell RNA sequencing identifies prolactin receptor as a therapeutic target in adenomyosis**

**Authors:** Runze Wang^1†^, Shuai Xu^2†^, Qionghua Cui^2†^, Xin Chen^3†^, Xuelian Wang^2^, Jinghao Liu^2^, Jie Liu^4^, Yuxuan Xin^2^, Hao Shen^2^, Fengxiang Lv^2^, Lan Zhu^3*^, Xinli Hu^2*^, Rui-Ping Xiao^1, 2, 5, 6, 7*^

Correspondence to***:***

xiaor@pku.edu.cn; huxxx025@pku.edu.cn; zhu_julie@vip.sina.com

**This file includes:**

Figures. S1 to S6

Tables S1 to S8.

**
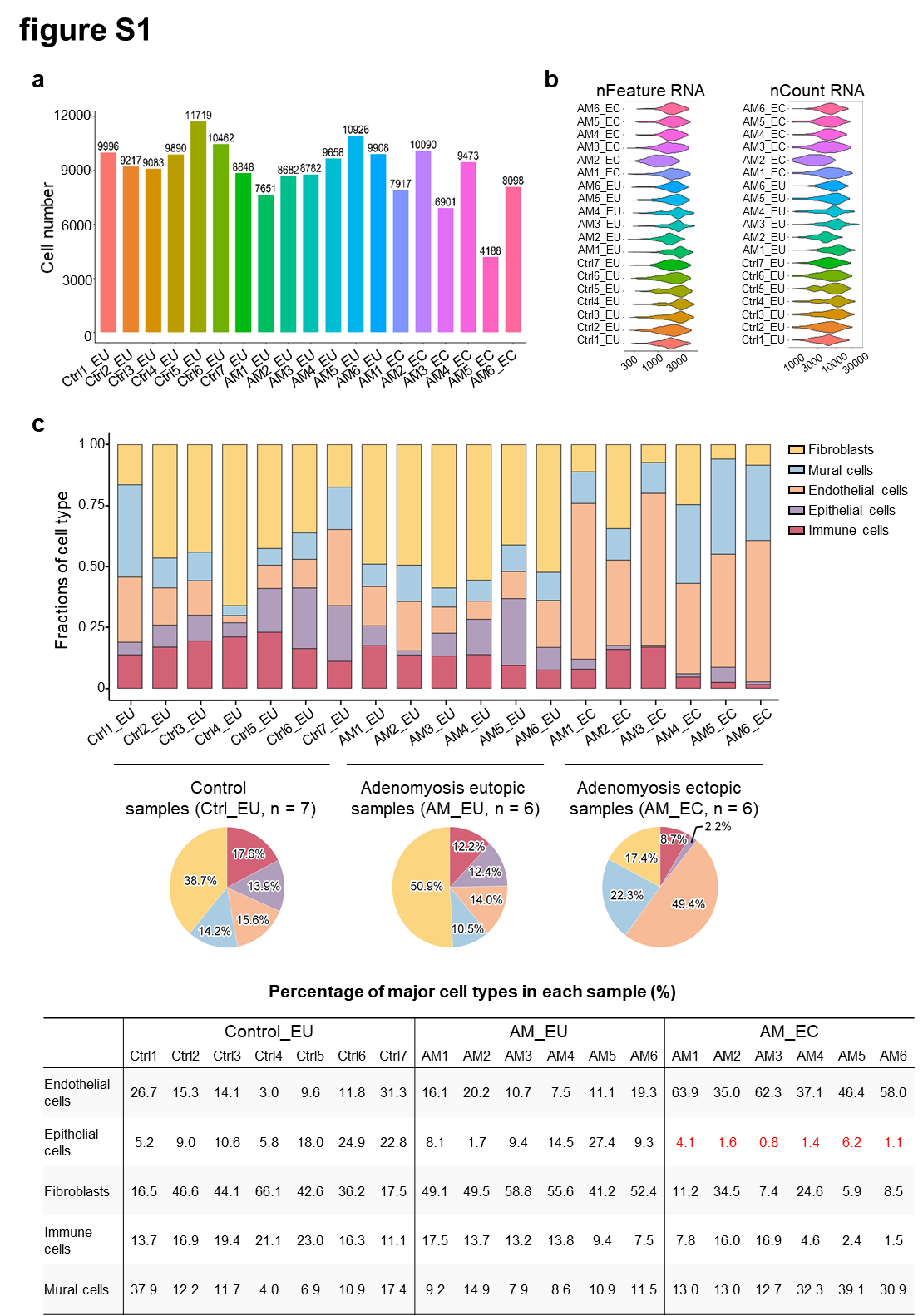
**

# Fig. S1. scRNA-seq of uterine biopsies of control participants and adenomyosis patients.

**a-b** Number of cells evaluated by scRNA-seq in each biopsy sample (**a**), and number of genes detected (nFeature_RNA) and total number of RNA molecules (nCount_RNA) per cell in each sample (**b**).

**c** Bar plot showing the fractions of major cell types in each biopsy sample. Pie charts showing the averaged proportions of the major cell types in the sample groups of control subjects (Ctrl_EU), eutopic (AM_EU) and ectopic (AM_EC) endometrial tissues of adenomyosis patients, with a table showing the numerical data of the percentages of the major cell types in each sample.


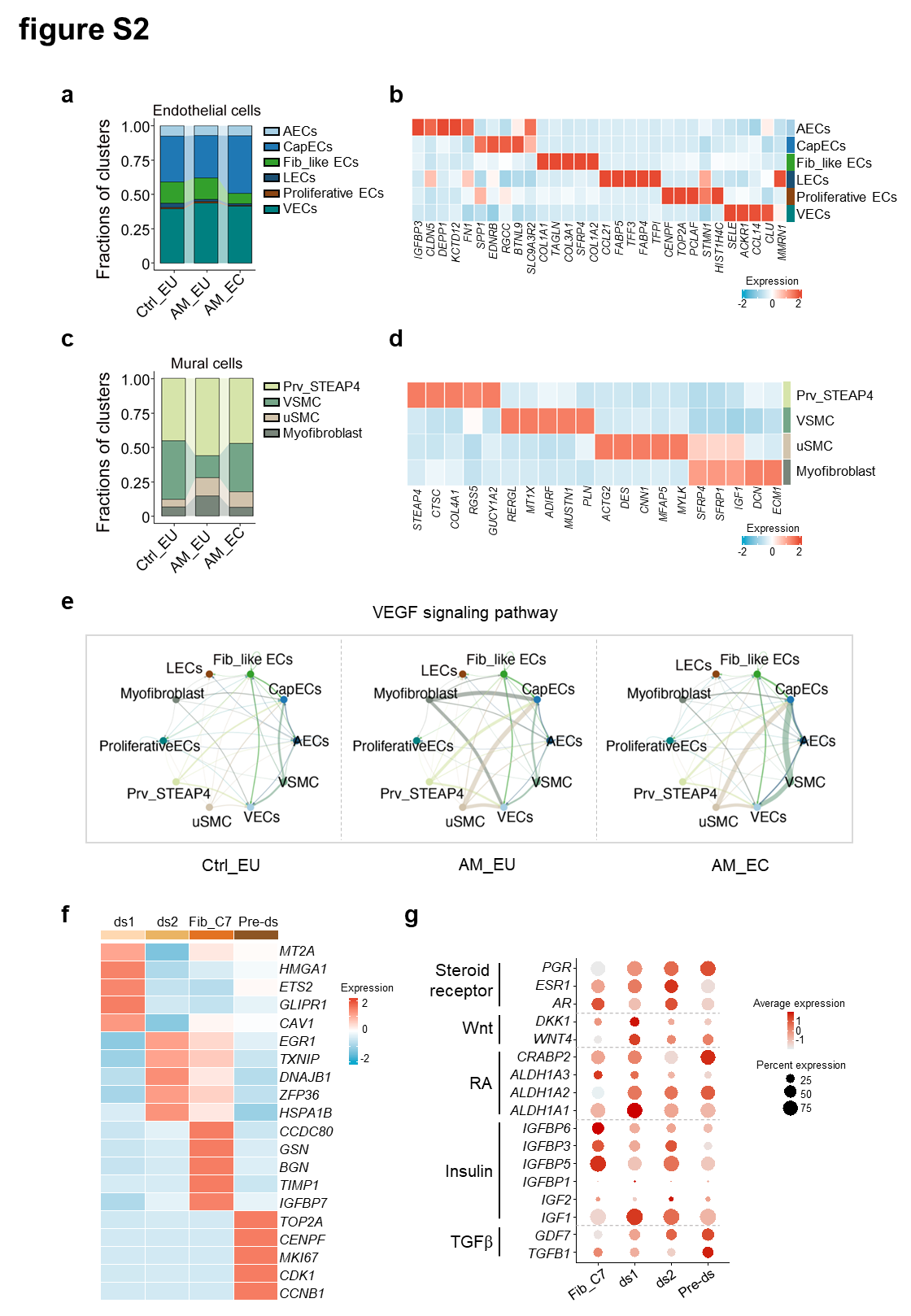


# Fig. S2. Subclustering of endothelial cells, mural cells, and fibroblasts.

**a** Fractions of endothelial cell subclusters in the control endometrium (Ctrl_EU), as well as eutopic endometrium (AM_EU) and lesions (AM_EC) of adenomyosis patients.

**b** Heatmap showing the expression levels of marker genes in each subcluster of endothelial cells.

**c** Fractions of mural cell subclusters in the control endometrium (Ctrl_EU), as well as eutopic endometrium (AM_EU) and lesions (AM_EC) of adenomyosis patients.

**d** Heatmap showing the expression levels of marker genes in each subcluster of mural cells.

**e** Circle plot showing the communications between subclusters of endothelial cells and mural cells via VEGF signaling. Edge color aligns with the source of signaling (sender), and edge width is proportional to the strength of interaction.

**f** Heatmap showing the expression levels of marker genes in each subcluster of fibroblasts.

**g** Dot plot showing Z score-scaled mean expression of genes coding steroid receptors and genes involved in WNT, retinoid acid (RA), insulin, and TGF-β signaling in fibroblast subclusters.

**
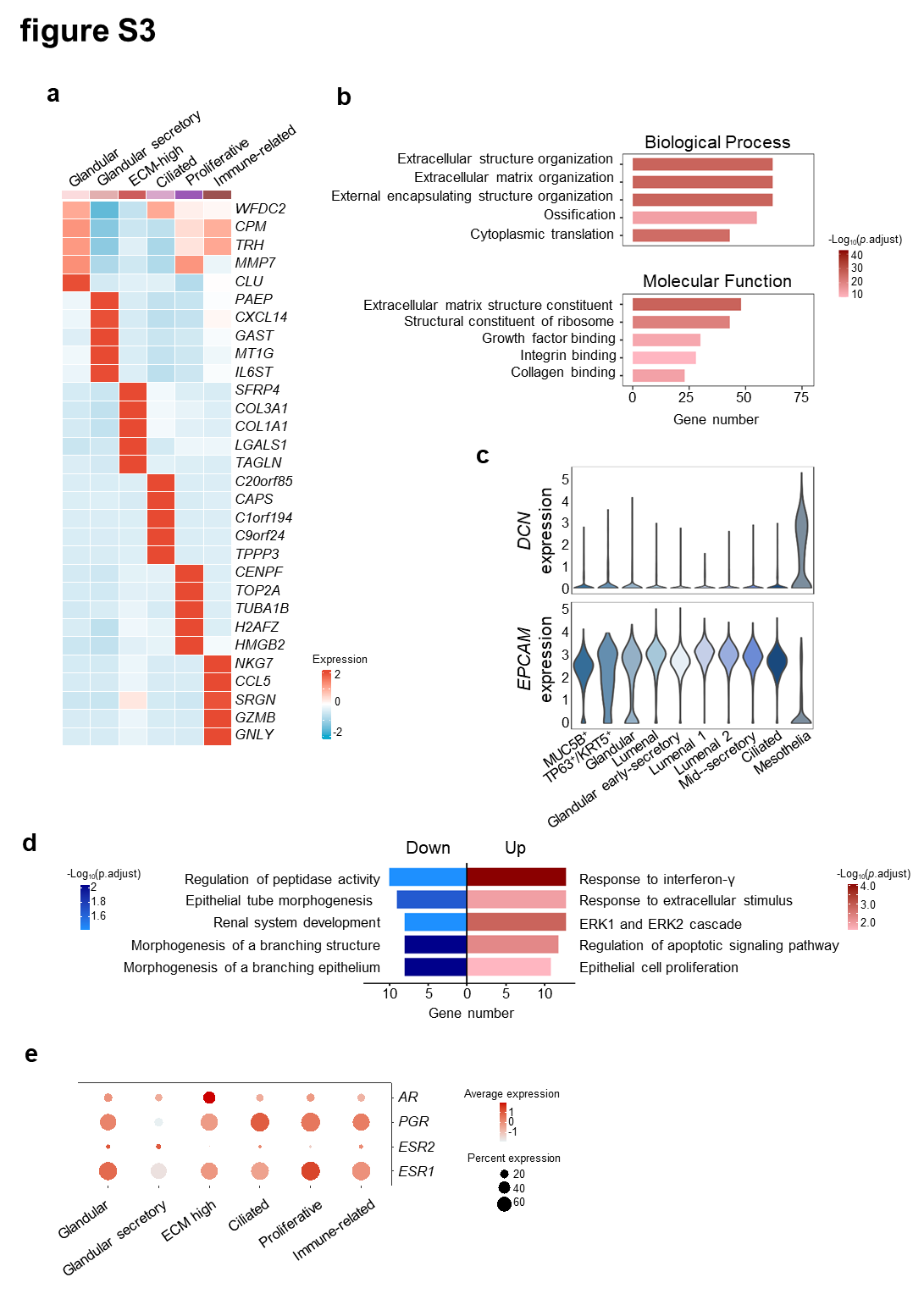
**

# Fig. S3. Subclustering of epithelial cells.

**a** Heatmap showing the expression levels of marker genes in each subcluster of epithelial cells.

**b** Bar plot showing the feature biological processes and molecular functions of ECM high epithelial cells enriched by Gene Ontology (GO) analysis.

**c** Violin plot showing the expression of DCN and EPCAM in the epithelial cells from scRNA-seq data by Tan et al ^21^.

**d** GO analysis enriched differential biological functions in ECM high epithelial cells from eutopic endometrium of adenomyosis patients relative to endometrium from control subjects.

**e** Dot plot showing Z score-scaled mean expression of genes coding steroid receptors in each subcluster of epithelial cells.


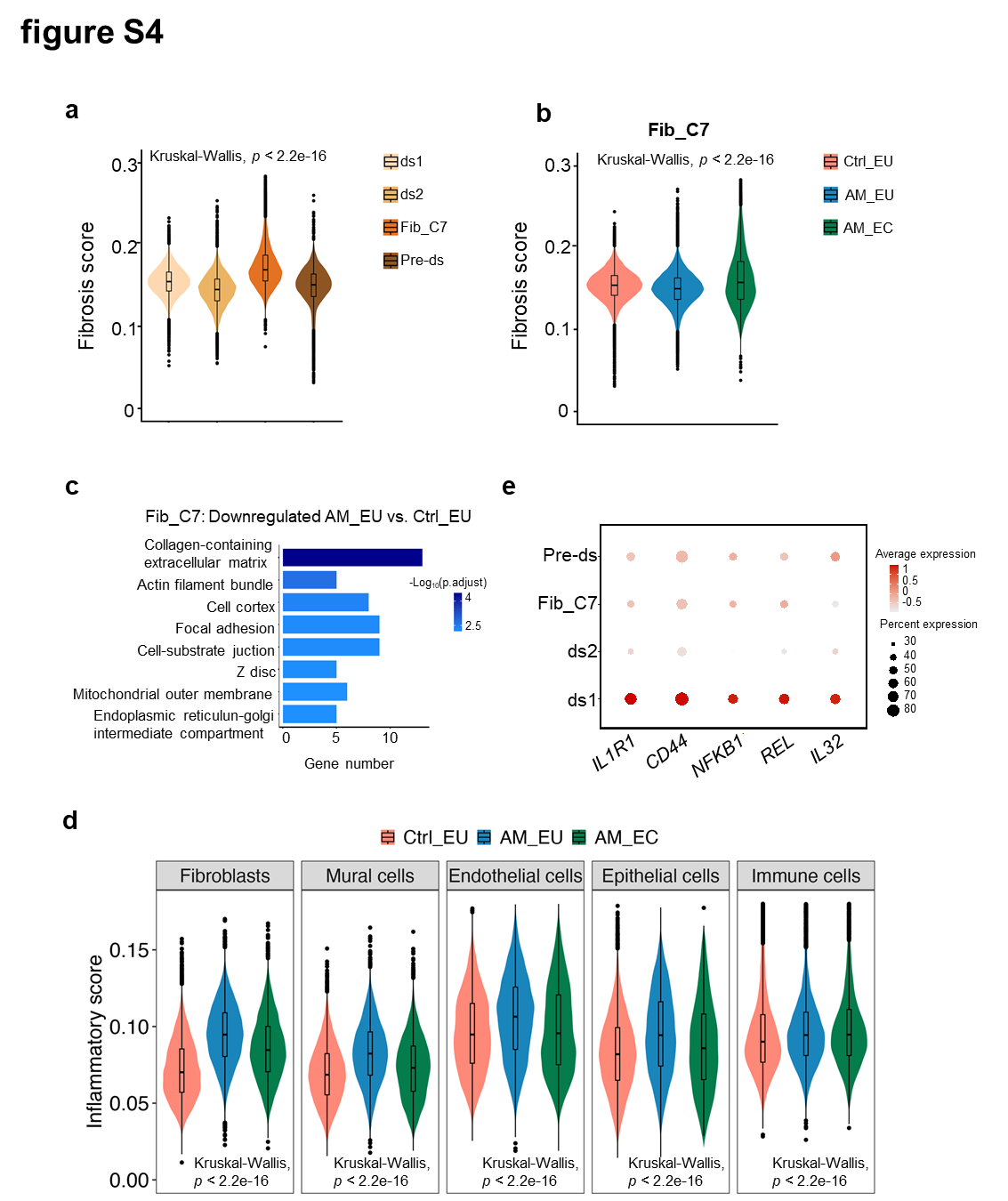


# Fig. S4. Enhanced fibrosis and inflammation in the fibroblasts of adenomyosis patients.

**a** Box and violin plots displaying the fibrosis scores across different fibroblast subclusters. The sample sizes were as follows: ds1: n = 24,924; ds2: n = 24,669; Fib_C7: n = 10,403; Pre-ds: n = 3,234.

**b** Box and violin plots displaying the fibrosis score of Fib_C7 in different sample groups. The sample sizes were as follows: Ctrl_EU (n = 4,135), AM_EU (n = 4,003), and AM_EC (n = 2,265).

**c** Bar plot showing the downregulated pathways in the Fib_C7 of AM_EU compared with those from Ctrl_EU enriched by Gene Ontology (GO) analysis.

**d** Box and violin plots showing the inflammatory scores of major cell types from different sample groups. The sample sizes were as follows: Ctrl_EU (Fibroblasts: n = 26,812; Mural cells: n = 9,858; Endothelial cells: n = 10,770; Epithelial cells: n = 9,618; Immune cells: n = 12,157), AM_EU (Fibroblasts: n = 28,284; Mural cells: n = 5,850; Endothelial cells: n = 7,775; Epithelial cells: n = 6,908; Immune cells: n = 6,790), and AM_EC (Fibroblasts: n = 8,134; Mural cells: n = 10,407; Endothelial cells: n = 23,039; Epithelial cells: n = 1,024; Immune cells: n = 4,063).

**e** Dot plot showing Z score-scaled mean expression of genes coding inflammation-related genes in the subclusters of fibroblasts.

In the box plots of panels **a**, **b** and **d**, the upper and lower quartiles are indicated, with horizontal lines representing the median. The whiskers extend to the range of data within 1.5 times the interquartile range above the third quartile and below the first quartile. Statistical differences were assessed using the Kruskal-Wallis test.

**
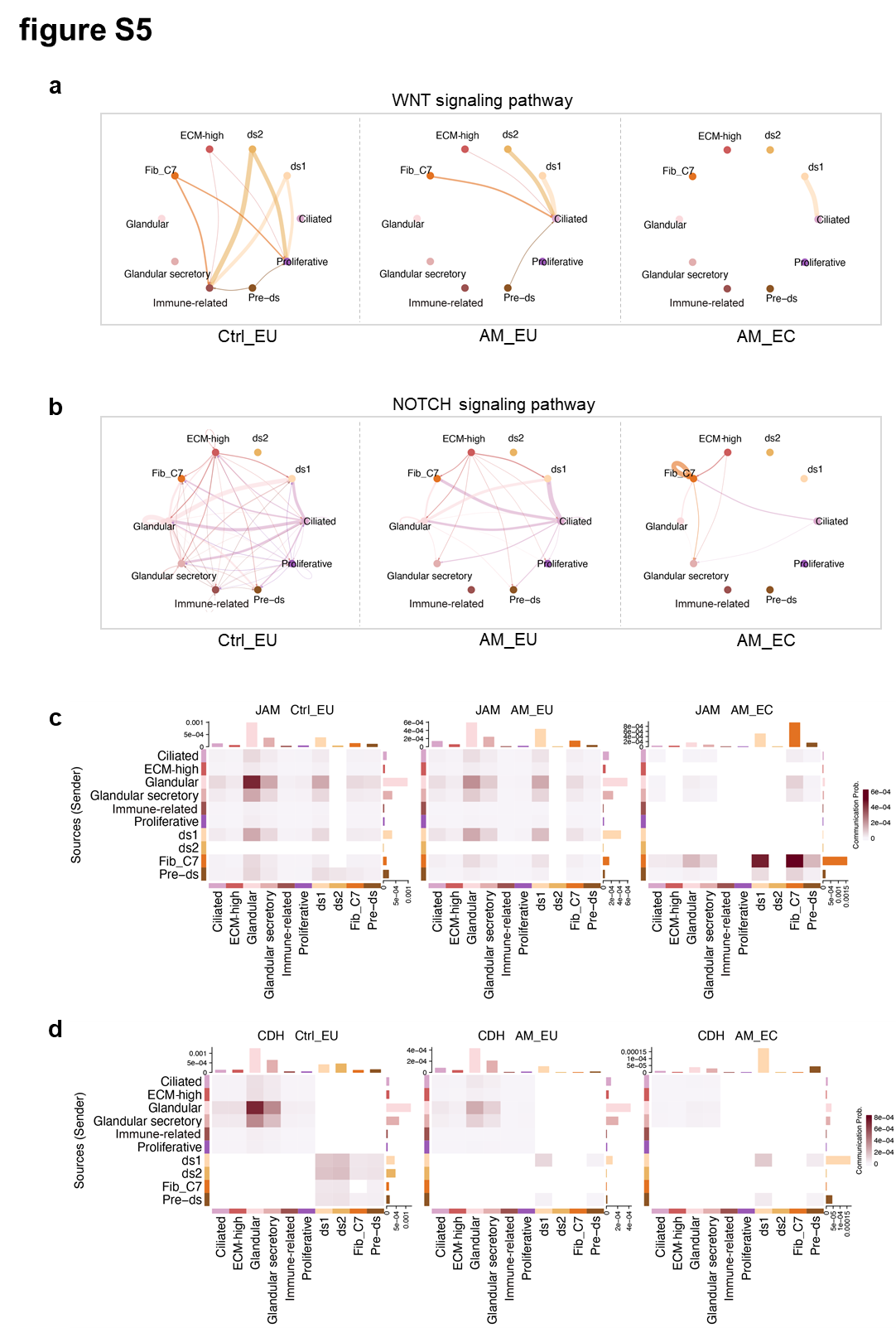
**

# Fig. S5. Impaired communications between fibroblasts and epithelial cells in adenomyosis.

**a-b** Circle plots showing the communications between subclusters of fibroblasts and epithelial cells via WNT (**a**) or NOTCH (**b**) signaling. Edge color aligns with the source of signaling (sender), and edge width is proportional to the strength of interaction.

**c-d** Heat maps showing the alterations in the interactions between fibroblasts and epithelial cell subclusters via JAM (**c**) or CDH (**d**) signaling.

**
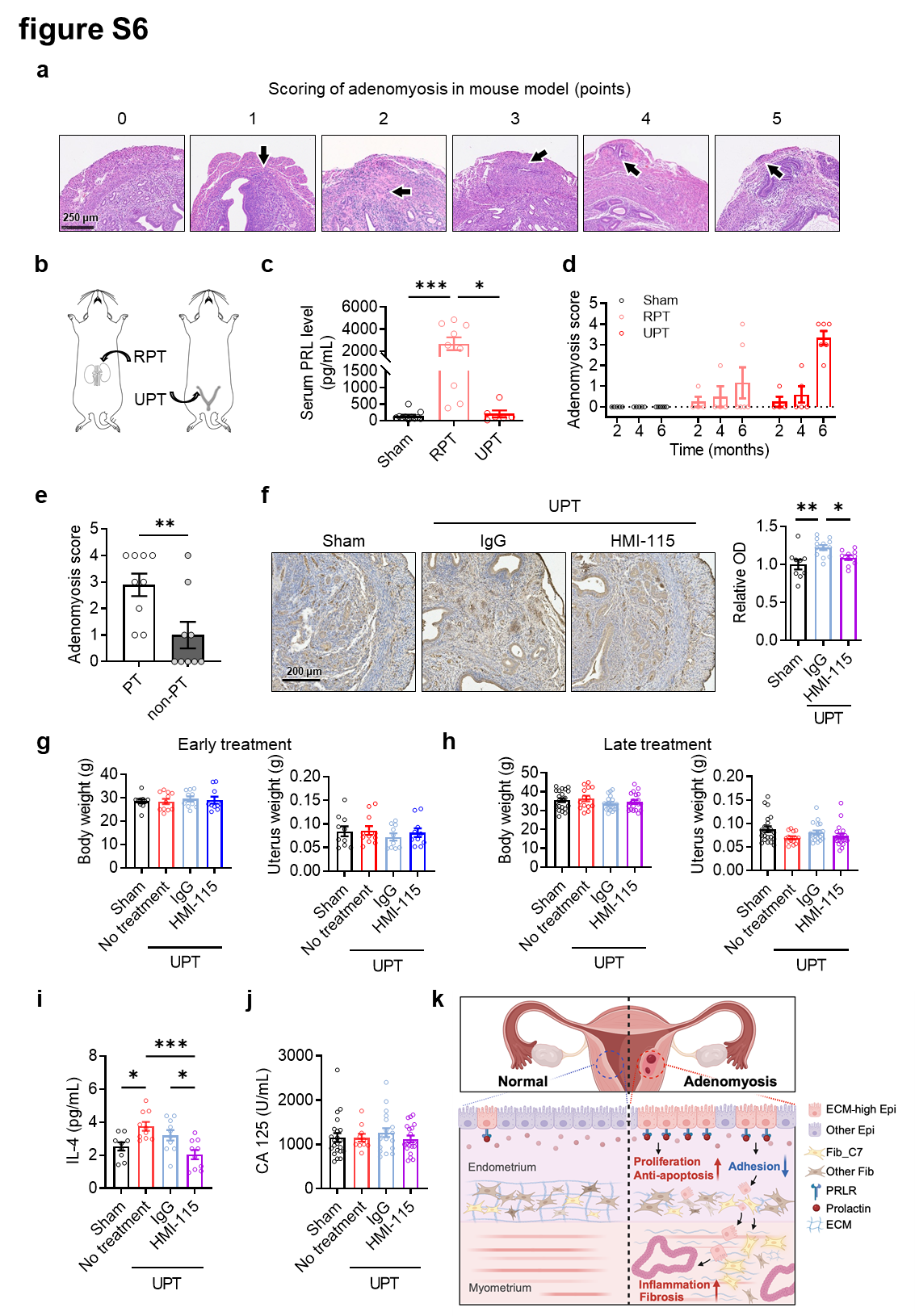
**

# Fig. S6. Increasing PRL induces whereas blocking PRLR mitigates adenomyosis in animal models.

**a** Images of uterine H&E staining illustrating the scoring scheme of adenomyosis. Arrows indicate lesions of ectopic endometrial glands. Scale bar, 250 μm.

**b** Diagram showing the transplantation of pituitary under the kidney capsule (RPT) or into the uterine horn (UPT).

**c-d** Serum PRL levels (Sham, n = 11; RPT, n = 9; UPT, n = 6) (**c**) and adenomyosis scores (**d**) of mice subjected to RPT or UPT (Sham, n = 5, 5, and 6; RPT, n = 4, 4, and 6; UPT, n = 4, 5, and 6 at 2, 4 and 6 months post transplantation surgery, respectively).

**e** The adenomyosis score of uterine side with (PT) or without (non-PT) pituitary transplant. (n = 9 for each group)

**f** Representative images and statistic results of the signal intensity of immunohistochemical staining of PRLR in the mice subjected to sham or UPT surgery and treated with human IgG or HMI-115. Scale bar, 200 μm. (Sham, n = 9; IgG, n =13; HMI-115, n = 10)

**g-h** Body weights and uterus weights of the mice subjected to early (**g**; Sham, n = 10; No treatment, n = 11; IgG, n = 10; HMI-115, n = 10) or late (**h**; Sham, n = 22; No treatment, n = 14; IgG, n = 19; HMI-115, n = 19) treatment with HMI-115.

**i-j** Serum levels of IL-4 (**i**; Sham, n = 9; No treatment, n = 10; IgG, n = 10; HMI-115, n = 10) or CA125 (**j**; Sham, n = 22; No treatment, n = 10; IgG, n = 19; HMI-115, n = 19) in mice subjected to sham or UPT surgery and treated with saline, human IgG, or HMI-115.

**k** Schematic diagram showing PRL signaling promotes adenomyosis via regulating epithelial proliferation and apoptosis. Image is generated using BioRender.

# Table S1. Patient information

| **ID** | **Group** | **Condition** | **Sample** | **Serum PRL (ng/mL)** |
| --- | --- | --- | --- | --- |
| Ctrl_1 | control | Endocervicitis | Endometrium (eutopic) | 21.2 |
| Ctrl_2 | control | Endocervicitis | Endometrium (eutopic) | — |
| Ctrl_3 | control | Endocervicitis | Endometrium (eutopic) | — |
| Ctrl_4 | control | Polyp of corpus uteri | Endometrium (eutopic) | — |
| Ctrl_5 | control | Endocervicitis | Endometrium (eutopic) | — |
| Ctrl_6 | control | Endocervicitis | Endometrium (eutopic) | — |
| Ctrl_7 | control | Endocervicitis | Endometrium (eutopic) | — |
| Ctrl_8 | control | Hysteromyoma | Serum | 11.5 |
| Ctrl_9 | control | Hysteromyoma | Serum | 6.7 |
| Ctrl_10 | control | Ovarian cyst | Serum | 38.7 |
| Ctrl_11 | control | Hysteromyoma | Serum | 9 |
| Ctrl_12 | control | Hysteromyoma | Serum | 14 |
| Ctrl_13 | control | Hysteromyoma | Serum | 10.4 |
| Ctrl_14 | control | Hysteromyoma | Serum | 13.1 |
| Ctrl_15 | control | Hysteromyoma | Serum | 10.1 |
| Ctrl_16 | control | Hysteromyoma | Serum | 19.9 |
| Ctrl_17 | control | Hysteromyoma | Serum | 14.2 |
| Ctrl_18 | control | Hysteromyoma | Serum | 15.3 |
| Ctrl_19 | control | Hysteromyoma | Serum | 3.9 |
| Ctrl_20 | control | Hysteromyoma | Serum | 7.0 |
| Ctrl_21 | control | Hysteromyoma | Serum | 6.8 |
| Ctrl_22 | control | Hysteromyoma | Serum | 10.4 |
| Ctrl_23 | control | Hysteromyoma | Serum | 7.5 |
| Ctrl_24 | control | Hysteromyoma | Serum | 17.4 |
| Ctrl_25 | control | Hysteromyoma | Serum | 12 |
| Ctrl_26 | control | Hysteromyoma | Serum | 14 |
| Ctrl_27 | control | Hysteromyoma | Serum | 6.8 |
| Ctrl_28 | control | Hysteromyoma | Serum | 20 |
| Ctrl_29 | control | Cervical lesions | Serum | 12.2 |
| AM_1 | case | Adenomyosis, endocervicitis | Endometrium (eutopic and ectopic) | 29.7 |
| AM_2 | case | Adenomyosis, endocervicitis | Endometrium (eutopic and ectopic) | — |
| AM_3 | case | Adenomyosis, endocervicitis | Endometrium (eutopic and ectopic) | — |
| AM_4 | case | Adenomyosis | Endometrium (eutopic and ectopic) | 7.6 |
| AM_5 | case | Adenomyosis | Endometrium (eutopic and ectopic) | — |
| AM_6 | case | Adenomyosis | Endometrium (eutopic and ectopic) | — |
| AM_7 | case | Adenomyosis | Serum | 17.3 |
| AM_8 | case | Adenomyosis | Serum | 4.8 |
| AM_9 | case | Adenomyosis | Serum | 9.8 |
| AM_10 | case | Adenomyosis | Serum | 15 |
| AM_11 | case | Adenomyosis | Serum | 9.3 |
| AM_12 | case | Adenomyosis | Serum | 13.5 |
| AM_13 | case | Adenomyosis | Serum | 14.0 |
| AM_14 | case | Adenomyosis | Serum | 8.1 |
| AM_15 | case | Adenomyosis | Serum | 17.8 |
| AM_16 | case | Adenomyosis | Serum | 6.1 |
| AM_17 | case | Adenomyosis | Serum | 9.8 |
| AM_18 | case | Adenomyosis | Serum | 17.1 |
| AM_19 | case | Adenomyosis | Serum | 10.8 |
| AM_20 | case | Adenomyosis | Serum | 7.6 |
| AM_21 | case | Adenomyosis | Serum | 22.2 |
| AM_22 | case | Adenomyosis | Serum | 7.7 |
| AM_23 | case | Adenomyosis | Serum | 10.0 |
| AM_24 | case | Adenomyosis | Serum | 10.3 |
| AM_25 | case | Adenomyosis | Serum | 6.5 |
| AM_26 | case | Adenomyosis | Serum | 15.6 |
| AM_27 | case | Adenomyosis | Serum | 30.5 |
| AM_28 | case | Adenomyosis | Serum | 6.0 |
| AM_29 | case | Adenomyosis | Serum | 6.2 |
| AM_30 | case | Adenomyosis | Serum | 12.0 |
| AM_31 | case | Adenomyosis | Serum | 6.7 |

# Table S2. Top 20 differentially expressed genes of major cell types

| **Gene Name** | **p_val** | **avg_log2FC** | **pct.1** | **pct.2** | **p_val_adj** | **Cell Type** |
| --- | --- | --- | --- | --- | --- | --- |
| *SFRP4* | 0 | 3.634011091 | 0.893 | 0.253 | 0 | Fibroblasts |
| *IGF1* | 0 | 2.994992855 | 0.901 | 0.179 | 0 | Fibroblasts |
| *DCN* | 0 | 2.974506642 | 0.961 | 0.192 | 0 | Fibroblasts |
| *COL1A1* | 0 | 2.968835945 | 0.991 | 0.422 | 0 | Fibroblasts |
| *MMP11* | 0 | 2.930820235 | 0.643 | 0.137 | 0 | Fibroblasts |
| *COL3A1* | 0 | 2.819233746 | 0.987 | 0.417 | 0 | Fibroblasts |
| *LUM* | 0 | 2.792718146 | 0.95 | 0.161 | 0 | Fibroblasts |
| *PTGDS* | 0 | 2.723981174 | 0.813 | 0.17 | 0 | Fibroblasts |
| *COL1A2* | 0 | 2.477275454 | 0.976 | 0.389 | 0 | Fibroblasts |
| *APOD* | 0 | 2.242021283 | 0.567 | 0.067 | 0 | Fibroblasts |
| *SFRP1* | 0 | 2.237610713 | 0.738 | 0.105 | 0 | Fibroblasts |
| *SERPINF1* | 0 | 2.201100168 | 0.892 | 0.162 | 0 | Fibroblasts |
| *MEG3* | 0 | 2.194665358 | 0.756 | 0.114 | 0 | Fibroblasts |
| *ECM1* | 0 | 2.13869013 | 0.77 | 0.123 | 0 | Fibroblasts |
| *OGN* | 0 | 2.093179293 | 0.716 | 0.08 | 0 | Fibroblasts |
| *RAMP1* | 0 | 2.044163451 | 0.917 | 0.206 | 0 | Fibroblasts |
| *COL6A1* | 0 | 1.879426979 | 0.957 | 0.36 | 0 | Fibroblasts |
| *PCOLCE* | 0 | 1.876386904 | 0.884 | 0.209 | 0 | Fibroblasts |
| *LGALS1* | 0 | 1.855636064 | 0.993 | 0.67 | 0 | Fibroblasts |
| *FBLN1* | 0 | 1.796990755 | 0.918 | 0.24 | 0 | Fibroblasts |
| *ACTA2* | 0 | 3.794790826 | 0.88 | 0.271 | 0 | Mural cells |
| *TAGLN* | 0 | 3.727879231 | 0.914 | 0.389 | 0 | Mural cells |
| *RGS5* | 0 | 3.632954527 | 0.671 | 0.066 | 0 | Mural cells |
| *MYH11* | 0 | 3.397429258 | 0.659 | 0.059 | 0 | Mural cells |
| *MYL9* | 0 | 3.015003691 | 0.939 | 0.463 | 0 | Mural cells |
| *TPM2* | 0 | 2.938492678 | 0.952 | 0.512 | 0 | Mural cells |
| *MUSTN1* | 0 | 2.868793154 | 0.466 | 0.021 | 0 | Mural cells |
| *ACTG2* | 0 | 2.773301062 | 0.32 | 0.026 | 0 | Mural cells |
| *ADIRF* | 0 | 2.719034256 | 0.754 | 0.366 | 0 | Mural cells |
| *MT1A* | 0 | 2.504472381 | 0.501 | 0.139 | 0 | Mural cells |
| *MYLK* | 0 | 2.36057107 | 0.789 | 0.229 | 0 | Mural cells |
| *CALD1* | 0 | 2.268197136 | 0.982 | 0.684 | 0 | Mural cells |
| *NDUFA4L2* | 0 | 2.224057582 | 0.496 | 0.028 | 0 | Mural cells |
| *RERGL* | 0 | 2.221973133 | 0.323 | 0.021 | 0 | Mural cells |
| *NOTCH3* | 0 | 2.220384471 | 0.72 | 0.09 | 0 | Mural cells |
| *CCL2* | 0 | 2.072285626 | 0.507 | 0.276 | 0 | Mural cells |
| *HIGD1B* | 0 | 2.028992558 | 0.47 | 0.021 | 0 | Mural cells |
| *TPM1* | 0 | 1.995452379 | 0.851 | 0.532 | 0 | Mural cells |
| *CNN1* | 0 | 1.993040881 | 0.474 | 0.111 | 0 | Mural cells |
| *GJA4* | 0 | 1.976491838 | 0.514 | 0.064 | 0 | Mural cells |
| *A2M* | 0 | 3.35434836 | 0.961 | 0.339 | 0 | Endothelial cells |
| *LIFR* | 0 | 3.352517473 | 0.859 | 0.114 | 0 | Endothelial cells |
| *ACKR1* | 0 | 3.218023539 | 0.512 | 0.026 | 0 | Endothelial cells |
| *IFI27* | 0 | 3.207522758 | 0.942 | 0.332 | 0 | Endothelial cells |
| *CCL14* | 0 | 3.171905271 | 0.541 | 0.019 | 0 | Endothelial cells |
| *CAVIN2* | 0 | 3.038700431 | 0.834 | 0.079 | 0 | Endothelial cells |
| *TM4SF1* | 0 | 3.018373675 | 0.878 | 0.205 | 0 | Endothelial cells |
| *EMCN* | 0 | 2.991623545 | 0.864 | 0.019 | 0 | Endothelial cells |
| *AQP1* | 0 | 2.914553515 | 0.716 | 0.032 | 0 | Endothelial cells |
| *RAMP2* | 0 | 2.81685215 | 0.852 | 0.189 | 0 | Endothelial cells |
| *FLT1* | 0 | 2.784393315 | 0.785 | 0.044 | 0 | Endothelial cells |
| *SELE* | 0 | 2.758553845 | 0.308 | 0.015 | 0 | Endothelial cells |
| *VWF* | 0 | 2.70495961 | 0.753 | 0.031 | 0 | Endothelial cells |
| *SPRY1* | 0 | 2.641043959 | 0.768 | 0.216 | 0 | Endothelial cells |
| *CALCRL* | 0 | 2.57640502 | 0.825 | 0.119 | 0 | Endothelial cells |
| *PLVAP* | 0 | 2.466681535 | 0.668 | 0.017 | 0 | Endothelial cells |
| *ADGRL4* | 0 | 2.416818708 | 0.789 | 0.021 | 0 | Endothelial cells |
| *GNG11* | 0 | 2.308056124 | 0.883 | 0.412 | 0 | Endothelial cells |
| *SLC9A3R2* | 0 | 2.297953585 | 0.667 | 0.237 | 0 | Endothelial cells |
| *EGFL7* | 0 | 2.28344761 | 0.698 | 0.056 | 0 | Endothelial cells |
| *PAEP* | 0 | 6.653494245 | 0.526 | 0.121 | 0 | Epithelial cells |
| *SCGB2A1* | 0 | 5.018692927 | 0.904 | 0.086 | 0 | Epithelial cells |
| *WFDC2* | 0 | 4.894715054 | 0.888 | 0.143 | 0 | Epithelial cells |
| *CLDN4* | 0 | 4.330107617 | 0.87 | 0.096 | 0 | Epithelial cells |
| *SLPI* | 0 | 4.042177241 | 0.809 | 0.041 | 0 | Epithelial cells |
| *CXCL14* | 0 | 3.983717221 | 0.41 | 0.039 | 0 | Epithelial cells |
| *ELF3* | 0 | 3.463675928 | 0.829 | 0.035 | 0 | Epithelial cells |
| *SCGB1D2* | 0 | 3.161544401 | 0.419 | 0.03 | 0 | Epithelial cells |
| *KRT18* | 0 | 3.121737415 | 0.881 | 0.092 | 0 | Epithelial cells |
| *CAPS* | 0 | 3.067160916 | 0.734 | 0.049 | 0 | Epithelial cells |
| *GPX3* | 0 | 3.047315351 | 0.484 | 0.098 | 0 | Epithelial cells |
| *CLDN3* | 0 | 2.89337491 | 0.805 | 0.032 | 0 | Epithelial cells |
| *KRT8* | 0 | 2.816418865 | 0.842 | 0.057 | 0 | Epithelial cells |
| *DEFB1* | 0 | 2.669232501 | 0.648 | 0.014 | 0 | Epithelial cells |
| *AGR2* | 0 | 2.593069449 | 0.678 | 0.015 | 0 | Epithelial cells |
| *UCA1* | 0 | 2.575039124 | 0.77 | 0.023 | 0 | Epithelial cells |
| *SPINT2* | 0 | 2.568837088 | 0.827 | 0.062 | 0 | Epithelial cells |
| *CD24* | 0 | 2.54455902 | 0.921 | 0.33 | 0 | Epithelial cells |
| *PIGR* | 0 | 2.514284126 | 0.645 | 0.014 | 0 | Epithelial cells |
| *ASRGL1* | 0 | 2.502173169 | 0.776 | 0.059 | 0 | Epithelial cells |
| *GNLY* | 0 | 5.489817785 | 0.268 | 0.072 | 0 | Immune cells |
| *CCL4* | 0 | 4.910707872 | 0.578 | 0.052 | 0 | Immune cells |
| *CCL5* | 0 | 4.226097124 | 0.551 | 0.024 | 0 | Immune cells |
| *CCL4L2* | 0 | 4.058712858 | 0.339 | 0.038 | 0 | Immune cells |
| *NKG7* | 0 | 4.058515776 | 0.459 | 0.018 | 0 | Immune cells |
| *SRGN* | 0 | 4.023727824 | 0.97 | 0.331 | 0 | Immune cells |
| *CCL3* | 0 | 3.909225472 | 0.295 | 0.035 | 0 | Immune cells |
| *CXCR4* | 0 | 3.867708924 | 0.744 | 0.092 | 0 | Immune cells |
| *CXCL8* | 0 | 3.639438256 | 0.321 | 0.217 | 0 | Immune cells |
| *PTPRC* | 0 | 3.575635918 | 0.815 | 0.011 | 0 | Immune cells |
| *TYROBP* | 0 | 3.179386457 | 0.523 | 0.015 | 0 | Immune cells |
| *GZMB* | 0 | 2.973928036 | 0.261 | 0.012 | 0 | Immune cells |
| *GZMA* | 0 | 2.965725819 | 0.426 | 0.009 | 0 | Immune cells |
| *GPR183* | 0 | 2.953434814 | 0.422 | 0.03 | 0 | Immune cells |
| *FCER1G* | 0 | 2.905730778 | 0.461 | 0.016 | 0 | Immune cells |
| *CST7* | 0 | 2.876565256 | 0.543 | 0.009 | 0 | Immune cells |
| *SAMSN1* | 0 | 2.850601602 | 0.614 | 0.009 | 0 | Immune cells |
| *HCST* | 0 | 2.787278523 | 0.617 | 0.014 | 0 | Immune cells |
| *RGS1* | 0 | 2.764531919 | 0.564 | 0.012 | 0 | Immune cells |
| *BCL2A1* | 0 | 2.70353728 | 0.309 | 0.015 | 0 | Immune cells |

# Table S3. Top 20 differentially expressed genes of epithelial cell subclusters

| **Gene Name** | **p_val** | **avg_log2FC** | **pct.1** | **pct.2** | **p_val_adj** | **Cell Type** |
| --- | --- | --- | --- | --- | --- | --- |
| *WFDC2* | 0 | 1.128636804 | 0.91 | 0.858 | 0 | Glandular |
| *SAT1* | 0 | 0.809978166 | 0.97 | 0.971 | 0 | Glandular |
| *SERPINA5* | 3.00E-306 | 0.860915001 | 0.429 | 0.168 | 8.14E-302 | Glandular |
| *CPM* | 7.41E-292 | 1.215750141 | 0.667 | 0.518 | 2.01E-287 | Glandular |
| *TRH* | 3.13E-239 | 1.16677758 | 0.348 | 0.138 | 8.48E-235 | Glandular |
| *MMP7* | 7.23E-185 | 1.302469759 | 0.405 | 0.218 | 1.96E-180 | Glandular |
| *MMP26* | 2.39E-184 | 0.925637718 | 0.379 | 0.189 | 6.48E-180 | Glandular |
| *CLU* | 3.19E-182 | 1.442873993 | 0.699 | 0.625 | 8.64E-178 | Glandular |
| *SLC26A2* | 1.47E-173 | 1.026513198 | 0.562 | 0.404 | 3.99E-169 | Glandular |
| *SCGB2A1* | 4.65E-159 | 0.879582829 | 0.907 | 0.899 | 1.26E-154 | Glandular |
| *SCGB1D2* | 1.44E-61 | 0.896721464 | 0.466 | 0.353 | 3.89E-57 | Glandular |
| *CXCL2* | 4.26E-32 | 0.873847498 | 0.477 | 0.442 | 1.16E-27 | Glandular |
| *PAEP* | 0 | 2.708911161 | 0.984 | 0.444 | 0 | Glandular secretory |
| *CXCL14* | 0 | 2.501177272 | 0.975 | 0.308 | 0 | Glandular secretory |
| *GAST* | 0 | 2.286308298 | 0.379 | 0.044 | 0 | Glandular secretory |
| *MT1G* | 0 | 2.12503988 | 0.847 | 0.287 | 0 | Glandular secretory |
| *IL6ST* | 0 | 2.064645459 | 0.979 | 0.685 | 0 | Glandular secretory |
| *GPX3* | 0 | 1.99830256 | 0.973 | 0.397 | 0 | Glandular secretory |
| *DPP4* | 0 | 1.907877687 | 0.829 | 0.128 | 0 | Glandular secretory |
| *VCAN* | 0 | 1.89510431 | 0.948 | 0.45 | 0 | Glandular secretory |
| *COMP* | 0 | 1.864296192 | 0.607 | 0.074 | 0 | Glandular secretory |
| *NUPR1* | 0 | 1.790946246 | 0.98 | 0.489 | 0 | Glandular secretory |
| *SLC1A1* | 0 | 1.789616892 | 0.907 | 0.173 | 0 | Glandular secretory |
| *SPP1* | 0 | 1.760546395 | 0.622 | 0.199 | 0 | Glandular secretory |
| *RIMKLB* | 0 | 1.711783578 | 0.984 | 0.484 | 0 | Glandular secretory |
| *MAOA* | 0 | 1.671924953 | 0.957 | 0.33 | 0 | Glandular secretory |
| *PLAAT3* | 0 | 1.634066761 | 0.965 | 0.447 | 0 | Glandular secretory |
| *DYNLT3* | 0 | 1.599842476 | 0.973 | 0.561 | 0 | Glandular secretory |
| *MT1F* | 0 | 1.586975239 | 0.889 | 0.386 | 0 | Glandular secretory |
| *SLC44A1* | 0 | 1.561377776 | 0.937 | 0.479 | 0 | Glandular secretory |
| *IDO1* | 0 | 1.51349833 | 0.966 | 0.343 | 0 | Glandular secretory |
| *G0S2* | 6.55E-273 | 1.596396302 | 0.587 | 0.273 | 1.78E-268 | Glandular secretory |
| *SFRP4* | 0 | 3.45274247 | 0.669 | 0.243 | 0 | ECM high |
| *COL3A1* | 0 | 2.971509063 | 0.873 | 0.312 | 0 | ECM high |
| *COL1A1* | 0 | 2.897429359 | 0.876 | 0.424 | 0 | ECM high |
| *LGALS1* | 0 | 2.596064612 | 0.918 | 0.513 | 0 | ECM high |
| *TAGLN* | 0 | 2.580528152 | 0.641 | 0.144 | 0 | ECM high |
| *SPARC* | 0 | 2.455388514 | 0.854 | 0.203 | 0 | ECM high |
| *DCN* | 0 | 2.434602505 | 0.724 | 0.107 | 0 | ECM high |
| *MGP* | 0 | 2.353324567 | 0.685 | 0.134 | 0 | ECM high |
| *C11orf96* | 0 | 2.251725804 | 0.659 | 0.186 | 0 | ECM high |
| *SPARCL1* | 0 | 2.237484209 | 0.805 | 0.152 | 0 | ECM high |
| *PTGDS* | 0 | 2.218022905 | 0.616 | 0.126 | 0 | ECM high |
| *MMP11* | 0 | 2.208496314 | 0.426 | 0.111 | 0 | ECM high |
| *LUM* | 0 | 2.168335573 | 0.669 | 0.096 | 0 | ECM high |
| *IGFBP5* | 0 | 2.040672843 | 0.656 | 0.226 | 0 | ECM high |
| *RAMP1* | 0 | 1.977919453 | 0.672 | 0.086 | 0 | ECM high |
| *IGF1* | 0 | 1.866144016 | 0.677 | 0.199 | 0 | ECM high |
| *TPM2* | 0 | 1.855921841 | 0.745 | 0.217 | 0 | ECM high |
| *COL6A1* | 0 | 1.784046055 | 0.726 | 0.142 | 0 | ECM high |
| *COL6A2* | 0 | 1.77629572 | 0.69 | 0.098 | 0 | ECM high |
| *ACTA2* | 0 | 1.76833865 | 0.508 | 0.098 | 0 | ECM high |
| *C20orf85* | 0 | 4.432902017 | 0.917 | 0.018 | 0 | Ciliated |
| *CAPS* | 0 | 4.040308695 | 0.979 | 0.714 | 0 | Ciliated |
| *C1orf194* | 0 | 4.03600239 | 0.905 | 0.027 | 0 | Ciliated |
| *C9orf24* | 0 | 3.988996148 | 0.867 | 0.019 | 0 | Ciliated |
| *TPPP3* | 0 | 3.976836642 | 0.895 | 0.058 | 0 | Ciliated |
| *RSPH1* | 0 | 3.909691799 | 0.913 | 0.073 | 0 | Ciliated |
| *C11orf88* | 0 | 3.730239843 | 0.854 | 0.013 | 0 | Ciliated |
| *AGR3* | 0 | 3.633338967 | 0.893 | 0.153 | 0 | Ciliated |
| *MORN2* | 0 | 3.469872011 | 0.894 | 0.326 | 0 | Ciliated |
| *MS4A8* | 0 | 3.394423745 | 0.758 | 0.043 | 0 | Ciliated |
| *PIFO* | 0 | 3.202834858 | 0.865 | 0.031 | 0 | Ciliated |
| *FAM183A* | 0 | 3.070001396 | 0.855 | 0.01 | 0 | Ciliated |
| *AL357093.2* | 0 | 3.066676393 | 0.816 | 0.01 | 0 | Ciliated |
| *CAPSL* | 0 | 3.017011239 | 0.84 | 0.009 | 0 | Ciliated |
| *SNTN* | 0 | 2.992891086 | 0.804 | 0.009 | 0 | Ciliated |
| *CETN2* | 0 | 2.85116853 | 0.881 | 0.426 | 0 | Ciliated |
| *OMG* | 0 | 2.830291452 | 0.731 | 0.008 | 0 | Ciliated |
| *C9orf116* | 0 | 2.825848646 | 0.848 | 0.161 | 0 | Ciliated |
| *C5orf49* | 0 | 2.774549822 | 0.812 | 0.023 | 0 | Ciliated |
| *PCAT19* | 0 | 2.49565164 | 0.741 | 0.134 | 0 | Ciliated |
| *CENPF* | 0 | 1.950995455 | 0.574 | 0.052 | 0 | Proliferative |
| *TOP2A* | 0 | 1.857590918 | 0.562 | 0.033 | 0 | Proliferative |
| *PCLAF* | 0 | 1.756552864 | 0.669 | 0.059 | 0 | Proliferative |
| *UBE2C* | 0 | 1.57294351 | 0.465 | 0.015 | 0 | Proliferative |
| *MKI67* | 0 | 1.50173806 | 0.529 | 0.024 | 0 | Proliferative |
| *NUSAP1* | 0 | 1.43837664 | 0.535 | 0.062 | 0 | Proliferative |
| *TK1* | 0 | 1.421219221 | 0.554 | 0.052 | 0 | Proliferative |
| *BIRC5* | 0 | 1.387845017 | 0.568 | 0.022 | 0 | Proliferative |
| *CCNB1* | 1.31E-304 | 1.576342903 | 0.57 | 0.086 | 3.54E-300 | Proliferative |
| *PTTG1* | 2.15E-262 | 1.719161564 | 0.564 | 0.099 | 5.82E-258 | Proliferative |
| *TUBA1B* | 1.95E-203 | 2.153992078 | 0.963 | 0.784 | 5.28E-199 | Proliferative |
| *CKS1B* | 1.98E-183 | 1.488957639 | 0.692 | 0.221 | 5.37E-179 | Proliferative |
| *H2AFZ* | 8.55E-182 | 1.973495082 | 0.929 | 0.763 | 2.32E-177 | Proliferative |
| *HMGN2* | 2.66E-167 | 1.793227844 | 0.929 | 0.8 | 7.21E-163 | Proliferative |
| *STMN1* | 4.22E-163 | 1.663556544 | 0.899 | 0.593 | 1.14E-158 | Proliferative |
| *HMGB2* | 2.21E-152 | 1.936377959 | 0.761 | 0.348 | 5.99E-148 | Proliferative |
| *TUBB* | 2.79E-141 | 1.723466972 | 0.888 | 0.649 | 7.56E-137 | Proliferative |
| *DUT* | 7.19E-114 | 1.559033649 | 0.767 | 0.438 | 1.95E-109 | Proliferative |
| *RCN1* | 2.25E-61 | 1.420669625 | 0.769 | 0.583 | 6.10E-57 | Proliferative |
| *HIST1H4C* | 9.90E-24 | 1.429397947 | 0.732 | 0.651 | 2.68E-19 | Proliferative |
| *NKG7* | 0 | 2.704196759 | 0.728 | 0.046 | 0 | Immune-related |
| *CCL5* | 0 | 2.588944226 | 0.751 | 0.053 | 0 | Immune-related |
| *GZMA* | 0 | 2.00711535 | 0.62 | 0.026 | 0 | Immune-related |
| *PTPRC* | 0 | 1.801775537 | 0.831 | 0.025 | 0 | Immune-related |
| *CST7* | 0 | 1.616653552 | 0.681 | 0.027 | 0 | Immune-related |
| *ARHGDIB* | 0 | 1.611664436 | 0.784 | 0.065 | 0 | Immune-related |
| *HCST* | 0 | 1.575884436 | 0.676 | 0.042 | 0 | Immune-related |
| *CD52* | 0 | 1.53883885 | 0.545 | 0.029 | 0 | Immune-related |
| *CTSW* | 0 | 1.463067671 | 0.516 | 0.02 | 0 | Immune-related |
| *CD7* | 0 | 1.401698623 | 0.592 | 0.035 | 0 | Immune-related |
| *RUNX3* | 0 | 1.301418025 | 0.624 | 0.019 | 0 | Immune-related |
| *SRGN* | 4.88E-297 | 2.592505493 | 0.953 | 0.126 | 1.32E-292 | Immune-related |
| *XCL2* | 1.61E-253 | 1.702127223 | 0.394 | 0.022 | 4.37E-249 | Immune-related |
| *GZMB* | 7.66E-212 | 2.193571843 | 0.437 | 0.033 | 2.08E-207 | Immune-related |
| *TYROBP* | 2.08E-177 | 1.415398367 | 0.441 | 0.041 | 5.64E-173 | Immune-related |
| *CCL4* | 4.38E-176 | 2.139282286 | 0.629 | 0.084 | 1.19E-171 | Immune-related |
| *TSC22D3* | 1.89E-100 | 1.581040491 | 0.789 | 0.246 | 5.11E-96 | Immune-related |
| *S100A4* | 3.83E-92 | 1.414954413 | 0.662 | 0.158 | 1.04E-87 | Immune-related |
| *IL32* | 1.04E-82 | 1.589570123 | 0.648 | 0.175 | 2.82E-78 | Immune-related |
| *GNLY* | 4.82E-63 | 4.472862483 | 0.493 | 0.134 | 1.31E-58 | Immune-related |

# Table S4. Top 20 differentially expressed genes of fibroblast subclusters

| **Gene Name** | **p_val** | **avg_log2FC** | **pct.1** | **pct.2** | **p_val_adj** | **Cell Type** |
| --- | --- | --- | --- | --- | --- | --- |
| PLIN2 | 0 | 1.07016809 | 0.696 | 0.43 | 0 | ds1 |
| MT2A | 0 | 1.01238921 | 0.722 | 0.525 | 0 | ds1 |
| SOD2 | 0 | 0.941715075 | 0.866 | 0.666 | 0 | ds1 |
| HMGA1 | 0 | 0.91675769 | 0.476 | 0.161 | 0 | ds1 |
| ETS2 | 0 | 0.864207329 | 0.824 | 0.487 | 0 | ds1 |
| IL32 | 0 | 0.83399157 | 0.638 | 0.389 | 0 | ds1 |
| GLIPR1 | 0 | 0.825110633 | 0.702 | 0.398 | 0 | ds1 |
| CAV1 | 0 | 0.815821253 | 0.712 | 0.412 | 0 | ds1 |
| SQSTM1 | 0 | 0.813270703 | 0.92 | 0.757 | 0 | ds1 |
| IL1R1 | 0 | 0.808702968 | 0.748 | 0.399 | 0 | ds1 |
| RBP1 | 0 | 0.806984892 | 0.935 | 0.762 | 0 | ds1 |
| CD44 | 0 | 0.788592817 | 0.882 | 0.631 | 0 | ds1 |
| ZFAND5 | 0 | 0.784083935 | 0.901 | 0.682 | 0 | ds1 |
| NAMPT | 0 | 0.747472967 | 0.827 | 0.557 | 0 | ds1 |
| DKK1 | 0 | 0.736987826 | 0.306 | 0.141 | 0 | ds1 |
| TNFRSF12A | 0 | 0.716679504 | 0.68 | 0.398 | 0 | ds1 |
| WTAP | 0 | 0.708475507 | 0.745 | 0.507 | 0 | ds1 |
| NFKB1 | 0 | 0.703414298 | 0.612 | 0.323 | 0 | ds1 |
| FAM107B | 0 | 0.685715917 | 0.512 | 0.258 | 0 | ds1 |
| PRDM1 | 0 | 0.680306943 | 0.648 | 0.363 | 0 | ds1 |
| FOS | 0 | 1.376074295 | 0.981 | 0.929 | 0 | ds2 |
| JUN | 0 | 1.113684247 | 0.97 | 0.918 | 0 | ds2 |
| EGR1 | 0 | 0.95268957 | 0.948 | 0.907 | 0 | ds2 |
| TXNIP | 0 | 0.950696107 | 0.847 | 0.677 | 0 | ds2 |
| DNAJB1 | 0 | 0.900881195 | 0.891 | 0.881 | 0 | ds2 |
| ZFP36 | 0 | 0.882052868 | 0.912 | 0.858 | 0 | ds2 |
| IER2 | 0 | 0.793958588 | 0.886 | 0.858 | 0 | ds2 |
| PTCH1 | 0 | 0.788660937 | 0.668 | 0.516 | 0 | ds2 |
| HSPA1B | 0 | 0.753064576 | 0.863 | 0.854 | 0 | ds2 |
| JUNB | 0 | 0.728418417 | 0.928 | 0.897 | 0 | ds2 |
| OSR2 | 0 | 0.667753533 | 0.704 | 0.555 | 0 | ds2 |
| HOXA10 | 0 | 0.667020486 | 0.637 | 0.473 | 0 | ds2 |
| DUSP1 | 0 | 0.628643929 | 0.866 | 0.846 | 0 | ds2 |
| IGFBP5 | 0 | 0.585942266 | 0.828 | 0.717 | 0 | ds2 |
| BTG2 | 4.67E-241 | 0.511601996 | 0.784 | 0.784 | 1.27E-236 | ds2 |
| KLK1 | 5.62E-217 | 0.5132749 | 0.275 | 0.185 | 1.52E-212 | ds2 |
| ID1 | 4.15E-210 | 0.510554692 | 0.607 | 0.535 | 1.12E-205 | ds2 |
| KLF2 | 1.21E-159 | 0.925232876 | 0.402 | 0.344 | 3.29E-155 | ds2 |
| IER5L | 4.90E-49 | 0.546286941 | 0.562 | 0.553 | 1.33E-44 | ds2 |
| GADD45G | 4.53E-29 | 0.617356222 | 0.516 | 0.53 | 1.23E-24 | ds2 |
| CCDC80 | 0 | 2.219350814 | 0.753 | 0.433 | 0 | Fib_C7 |
| GSN | 0 | 1.730225576 | 0.854 | 0.68 | 0 | Fib_C7 |
| BGN | 0 | 1.630815519 | 0.635 | 0.244 | 0 | Fib_C7 |
| TIMP1 | 0 | 1.484132458 | 0.975 | 0.914 | 0 | Fib_C7 |
| IGFBP7 | 0 | 1.434440564 | 0.953 | 0.872 | 0 | Fib_C7 |
| PLAC9 | 0 | 1.345002931 | 0.449 | 0.195 | 0 | Fib_C7 |
| ASPN | 0 | 1.315545836 | 0.349 | 0.044 | 0 | Fib_C7 |
| DCN | 0 | 1.236959822 | 0.988 | 0.955 | 0 | Fib_C7 |
| CST3 | 0 | 1.162555401 | 0.917 | 0.812 | 0 | Fib_C7 |
| A2M | 0 | 1.137391634 | 0.531 | 0.252 | 0 | Fib_C7 |
| SPARCL1 | 0 | 1.131166751 | 0.979 | 0.939 | 0 | Fib_C7 |
| CCL2 | 0 | 1.124195004 | 0.419 | 0.253 | 0 | Fib_C7 |
| ADIRF | 0 | 1.068862194 | 0.441 | 0.193 | 0 | Fib_C7 |
| HES1 | 0 | 1.055705412 | 0.539 | 0.247 | 0 | Fib_C7 |
| IGFBP5 | 0 | 1.047727455 | 0.875 | 0.738 | 0 | Fib_C7 |
| IGFBP6 | 0 | 1.036726239 | 0.494 | 0.317 | 0 | Fib_C7 |
| FRZB | 0 | 1.019781634 | 0.284 | 0.041 | 0 | Fib_C7 |
| APOD | 4.76E-275 | 0.985707257 | 0.67 | 0.547 | 1.29E-270 | Fib_C7 |
| MGP | 2.06E-238 | 1.272475737 | 0.821 | 0.73 | 5.59E-234 | Fib_C7 |
| CFD | 1.56E-222 | 1.069743508 | 0.368 | 0.231 | 4.23E-218 | Fib_C7 |
| HIST1H4C | 0 | 2.984830501 | 0.792 | 0.498 | 0 | Pre-ds |
| TOP2A | 0 | 2.924984598 | 0.671 | 0.022 | 0 | Pre-ds |
| CENPF | 0 | 2.762751654 | 0.676 | 0.05 | 0 | Pre-ds |
| HMGB2 | 0 | 2.573937683 | 0.856 | 0.391 | 0 | Pre-ds |
| MKI67 | 0 | 2.339298584 | 0.575 | 0.01 | 0 | Pre-ds |
| PCLAF | 0 | 2.284471625 | 0.774 | 0.04 | 0 | Pre-ds |
| TUBA1B | 0 | 2.213975902 | 0.954 | 0.818 | 0 | Pre-ds |
| STMN1 | 0 | 2.177383283 | 0.911 | 0.558 | 0 | Pre-ds |
| NUSAP1 | 0 | 2.028061089 | 0.64 | 0.034 | 0 | Pre-ds |
| TYMS | 0 | 1.96725117 | 0.721 | 0.035 | 0 | Pre-ds |
| PTTG1 | 0 | 1.835188642 | 0.554 | 0.055 | 0 | Pre-ds |
| TPX2 | 0 | 1.770647399 | 0.577 | 0.014 | 0 | Pre-ds |
| UBE2C | 0 | 1.763842751 | 0.543 | 0.01 | 0 | Pre-ds |
| SMC4 | 0 | 1.724863627 | 0.712 | 0.142 | 0 | Pre-ds |
| TUBB | 0 | 1.702164419 | 0.932 | 0.813 | 0 | Pre-ds |
| H2AFZ | 0 | 1.584517118 | 0.942 | 0.808 | 0 | Pre-ds |
| ASPM | 0 | 1.576237597 | 0.454 | 0.005 | 0 | Pre-ds |
| HMGN2 | 0 | 1.572397261 | 0.947 | 0.873 | 0 | Pre-ds |
| DEK | 0 | 1.55652352 | 0.879 | 0.591 | 0 | Pre-ds |
| HMGB1 | 0 | 1.498819589 | 0.992 | 0.975 | 0 | Pre-ds |

# Table S5. Top 20 differentially expressed genes of endothelial cell subclusters

| **Gene Name** | **p_val** | **avg_log2FC** | **pct.1** | **pct.2** | **p_val_adj** | **Cell Type** |
| --- | --- | --- | --- | --- | --- | --- |
| SPP1 | 0 | 1.829619142 | 0.287 | 0.092 | 0 | CapECs |
| EDNRB | 0 | 1.587390376 | 0.388 | 0.15 | 0 | CapECs |
| RGCC | 0 | 1.471556595 | 0.637 | 0.493 | 0 | CapECs |
| BTNL9 | 0 | 1.339999991 | 0.343 | 0.089 | 0 | CapECs |
| SLC9A3R2 | 0 | 1.180312588 | 0.781 | 0.603 | 0 | CapECs |
| DDIT4 | 0 | 1.119168955 | 0.627 | 0.42 | 0 | CapECs |
| PLVAP | 0 | 1.094465542 | 0.761 | 0.615 | 0 | CapECs |
| KDR | 0 | 1.023933142 | 0.639 | 0.511 | 0 | CapECs |
| FLT1 | 0 | 0.976617359 | 0.83 | 0.76 | 0 | CapECs |
| RBP7 | 0 | 0.959945321 | 0.463 | 0.317 | 0 | CapECs |
| TIMP3 | 0 | 0.951129934 | 0.902 | 0.788 | 0 | CapECs |
| UACA | 0 | 0.941822523 | 0.721 | 0.609 | 0 | CapECs |
| PODXL | 0 | 0.939552847 | 0.793 | 0.647 | 0 | CapECs |
| COL15A1 | 0 | 0.932812749 | 0.572 | 0.394 | 0 | CapECs |
| INSR | 0 | 0.930378795 | 0.685 | 0.635 | 0 | CapECs |
| ACE | 0 | 0.923105405 | 0.402 | 0.207 | 0 | CapECs |
| GSN | 0 | 0.880473168 | 0.909 | 0.858 | 0 | CapECs |
| CD34 | 0 | 0.874334426 | 0.781 | 0.667 | 0 | CapECs |
| ID2 | 0 | 0.872066309 | 0.642 | 0.548 | 0 | CapECs |
| PLPP1 | 7.74E-252 | 0.898437341 | 0.581 | 0.504 | 2.10E-247 | CapECs |
| SELE | 0 | 2.618545388 | 0.539 | 0.144 | 0 | VECs |
| ACKR1 | 0 | 2.405737516 | 0.843 | 0.281 | 0 | VECs |
| CCL14 | 0 | 1.786620077 | 0.789 | 0.369 | 0 | VECs |
| CLU | 0 | 1.766299035 | 0.879 | 0.494 | 0 | VECs |
| MMRN1 | 0 | 1.371260393 | 0.609 | 0.15 | 0 | VECs |
| VCAM1 | 0 | 1.296220344 | 0.356 | 0.092 | 0 | VECs |
| SOD2 | 0 | 1.183179303 | 0.736 | 0.537 | 0 | VECs |
| IL1R1 | 0 | 1.037489386 | 0.583 | 0.179 | 0 | VECs |
| POSTN | 0 | 1.004818065 | 0.329 | 0.086 | 0 | VECs |
| SELP | 0 | 0.999069251 | 0.498 | 0.103 | 0 | VECs |
| CNKSR3 | 0 | 0.980163544 | 0.524 | 0.22 | 0 | VECs |
| TM4SF1 | 0 | 0.971922565 | 0.941 | 0.835 | 0 | VECs |
| ICAM1 | 0 | 0.945274695 | 0.677 | 0.455 | 0 | VECs |
| HLA-DRA | 0 | 0.937853942 | 0.916 | 0.661 | 0 | VECs |
| NNMT | 0 | 0.917214896 | 0.742 | 0.476 | 0 | VECs |
| GPM6A | 0 | 0.877908097 | 0.383 | 0.062 | 0 | VECs |
| ADIRF | 0 | 0.857527449 | 0.895 | 0.67 | 0 | VECs |
| PRCP | 0 | 0.851988611 | 0.702 | 0.364 | 0 | VECs |
| ACTN1 | 0 | 0.838235259 | 0.554 | 0.21 | 0 | VECs |
| CCL2 | 3.34E-244 | 0.915049216 | 0.509 | 0.359 | 9.06E-240 | VECs |
| COL1A1 | 0 | 3.461164234 | 0.785 | 0.161 | 0 | Fib_like ECs |
| TAGLN | 0 | 3.166806026 | 0.666 | 0.212 | 0 | Fib_like ECs |
| COL3A1 | 0 | 3.158427992 | 0.791 | 0.202 | 0 | Fib_like ECs |
| SFRP4 | 0 | 3.108379325 | 0.512 | 0.113 | 0 | Fib_like ECs |
| COL1A2 | 0 | 3.052081347 | 0.763 | 0.114 | 0 | Fib_like ECs |
| ACTA2 | 0 | 2.905164328 | 0.562 | 0.113 | 0 | Fib_like ECs |
| IGFBP5 | 0 | 2.754756739 | 0.668 | 0.126 | 0 | Fib_like ECs |
| TPM2 | 0 | 2.557670484 | 0.74 | 0.179 | 0 | Fib_like ECs |
| RGS5 | 0 | 2.546385679 | 0.448 | 0.093 | 0 | Fib_like ECs |
| LGALS1 | 0 | 2.376787444 | 0.897 | 0.505 | 0 | Fib_like ECs |
| DCN | 0 | 2.340928382 | 0.538 | 0.062 | 0 | Fib_like ECs |
| LUM | 0 | 2.188047586 | 0.487 | 0.054 | 0 | Fib_like ECs |
| MYL9 | 0 | 2.011054806 | 0.706 | 0.316 | 0 | Fib_like ECs |
| IGF1 | 0 | 1.921348302 | 0.429 | 0.052 | 0 | Fib_like ECs |
| MMP11 | 0 | 1.88004323 | 0.327 | 0.053 | 0 | Fib_like ECs |
| TPM1 | 0 | 1.55901152 | 0.695 | 0.303 | 0 | Fib_like ECs |
| PTGDS | 0 | 1.558626783 | 0.373 | 0.133 | 0 | Fib_like ECs |
| RAMP1 | 0 | 1.549003778 | 0.449 | 0.03 | 0 | Fib_like ECs |
| MYH11 | 0 | 1.522757255 | 0.308 | 0.054 | 0 | Fib_like ECs |
| COL6A3 | 0 | 1.522430348 | 0.498 | 0.025 | 0 | Fib_like ECs |
| IGFBP3 | 0 | 3.078609107 | 0.689 | 0.111 | 0 | AECs |
| CLDN5 | 0 | 2.3624441 | 0.866 | 0.453 | 0 | AECs |
| DEPP1 | 0 | 2.175381801 | 0.639 | 0.197 | 0 | AECs |
| KCTD12 | 0 | 2.173996004 | 0.899 | 0.508 | 0 | AECs |
| FN1 | 0 | 2.008887926 | 0.823 | 0.316 | 0 | AECs |
| FBLN5 | 0 | 1.961687739 | 0.575 | 0.036 | 0 | AECs |
| ABI3BP | 0 | 1.780707149 | 0.523 | 0.123 | 0 | AECs |
| FBLN2 | 0 | 1.774125178 | 0.521 | 0.068 | 0 | AECs |
| SRP14 | 0 | 1.740668976 | 0.993 | 0.943 | 0 | AECs |
| SEMA3G | 0 | 1.730930808 | 0.613 | 0.03 | 0 | AECs |
| GJA5 | 0 | 1.646010811 | 0.437 | 0.017 | 0 | AECs |
| ADAMTS1 | 0 | 1.603360669 | 0.805 | 0.492 | 0 | AECs |
| JAG1 | 0 | 1.590899638 | 0.863 | 0.557 | 0 | AECs |
| C1QTNF3 | 0 | 1.506042433 | 0.278 | 0.03 | 0 | AECs |
| CXCL12 | 0 | 1.499339635 | 0.611 | 0.143 | 0 | AECs |
| SULF1 | 0 | 1.492770504 | 0.438 | 0.074 | 0 | AECs |
| SRGN | 0 | 1.429500656 | 0.981 | 0.83 | 0 | AECs |
| SERPINE2 | 0 | 1.429224199 | 0.38 | 0.024 | 0 | AECs |
| LTBP1 | 0 | 1.419330842 | 0.654 | 0.133 | 0 | AECs |
| CXCL2 | 8.69E-192 | 1.425621638 | 0.443 | 0.226 | 2.36E-187 | AECs |
| CCL21 | 0 | 6.993619935 | 0.982 | 0.011 | 0 | LECs |
| FABP5 | 0 | 4.167268084 | 0.815 | 0.064 | 0 | LECs |
| TFF3 | 0 | 3.762201903 | 0.839 | 0.005 | 0 | LECs |
| FABP4 | 0 | 3.235852714 | 0.395 | 0.005 | 0 | LECs |
| TFPI | 0 | 3.197404813 | 0.963 | 0.368 | 0 | LECs |
| PDPN | 0 | 2.620875339 | 0.759 | 0.019 | 0 | LECs |
| PROX1 | 0 | 2.355568177 | 0.702 | 0.104 | 0 | LECs |
| NRP2 | 0 | 2.328363249 | 0.788 | 0.215 | 0 | LECs |
| NR2F1 | 0 | 2.31812666 | 0.695 | 0.104 | 0 | LECs |
| MAF | 0 | 2.187234681 | 0.671 | 0.07 | 0 | LECs |
| MMRN1 | 0 | 2.13904702 | 0.865 | 0.33 | 0 | LECs |
| MRC1 | 0 | 2.12206075 | 0.661 | 0.028 | 0 | LECs |
| GYPC | 0 | 2.061329662 | 0.777 | 0.301 | 0 | LECs |
| LAMA4 | 0 | 1.827015011 | 0.776 | 0.253 | 0 | LECs |
| SEMA3D | 0 | 1.82187842 | 0.589 | 0.045 | 0 | LECs |
| PPFIBP1 | 1.08E-305 | 2.068794521 | 0.898 | 0.531 | 2.93E-301 | LECs |
| ANGPT2 | 3.13E-258 | 2.017325405 | 0.774 | 0.306 | 8.48E-254 | LECs |
| CXCL1 | 2.47E-254 | 2.130072718 | 0.302 | 0.043 | 6.69E-250 | LECs |
| AKAP12 | 3.37E-230 | 2.403640337 | 0.631 | 0.208 | 9.15E-226 | LECs |
| HSPA6 | 4.37E-111 | 2.210653177 | 0.287 | 0.076 | 1.18E-106 | LECs |
| CENPF | 0 | 2.198589889 | 0.519 | 0.034 | 0 | ProliferativeECs |
| TOP2A | 0 | 2.057008073 | 0.546 | 0.019 | 0 | ProliferativeECs |
| PCLAF | 0 | 1.815765423 | 0.62 | 0.023 | 0 | ProliferativeECs |
| TYMS | 0 | 1.751904177 | 0.651 | 0.026 | 0 | ProliferativeECs |
| MKI67 | 0 | 1.675066422 | 0.492 | 0.009 | 0 | ProliferativeECs |
| NUSAP1 | 0 | 1.662762321 | 0.546 | 0.015 | 0 | ProliferativeECs |
| TK1 | 0 | 1.529883835 | 0.597 | 0.033 | 0 | ProliferativeECs |
| TPX2 | 0 | 1.270755742 | 0.437 | 0.011 | 0 | ProliferativeECs |
| ASPM | 0 | 1.230155502 | 0.369 | 0.007 | 0 | ProliferativeECs |
| PTTG1 | 1.06E-205 | 1.347929159 | 0.414 | 0.044 | 2.86E-201 | ProliferativeECs |
| SMC4 | 9.66E-120 | 1.407856761 | 0.705 | 0.211 | 2.62E-115 | ProliferativeECs |
| STMN1 | 3.68E-108 | 2.052187526 | 0.817 | 0.368 | 9.99E-104 | ProliferativeECs |
| PCNA | 2.89E-101 | 1.212020879 | 0.573 | 0.154 | 7.83E-97 | ProliferativeECs |
| HMGB2 | 1.43E-83 | 1.704784286 | 0.824 | 0.437 | 3.87E-79 | ProliferativeECs |
| TUBA1B | 1.02E-79 | 1.562704604 | 0.963 | 0.803 | 2.76E-75 | ProliferativeECs |
| HMGN2 | 5.88E-79 | 1.759809398 | 0.892 | 0.586 | 1.59E-74 | ProliferativeECs |
| TUBB | 1.03E-77 | 1.655558379 | 0.892 | 0.604 | 2.78E-73 | ProliferativeECs |
| H2AFZ | 4.20E-75 | 1.530031017 | 0.919 | 0.698 | 1.14E-70 | ProliferativeECs |
| DUT | 5.75E-56 | 1.247666719 | 0.766 | 0.399 | 1.56E-51 | ProliferativeECs |
| HIST1H4C | 2.00E-21 | 2.150286175 | 0.637 | 0.456 | 5.41E-17 | ProliferativeECs |

# Table S6. Top 20 differentially expressed genes of immune cell subclusters

| **Gene Name** | **p_val** | **avg_log2FC** | **pct.1** | **pct.2** | **p_val_adj** | **Cell Type** |
| --- | --- | --- | --- | --- | --- | --- |
| IL7R | 0 | 2.849103652 | 0.472 | 0.091 | 0 | T cells |
| CD3D | 0 | 2.59417265 | 0.734 | 0.054 | 0 | T cells |
| CD3G | 0 | 2.122756915 | 0.572 | 0.036 | 0 | T cells |
| TRBC2 | 0 | 2.06376751 | 0.67 | 0.23 | 0 | T cells |
| CD2 | 0 | 2.063602951 | 0.673 | 0.16 | 0 | T cells |
| TRAC | 0 | 1.983428397 | 0.521 | 0.056 | 0 | T cells |
| GZMK | 0 | 1.864075797 | 0.467 | 0.16 | 0 | T cells |
| BCL11B | 0 | 1.783740844 | 0.446 | 0.045 | 0 | T cells |
| IL32 | 0 | 1.771828376 | 0.796 | 0.258 | 0 | T cells |
| SPOCK2 | 0 | 1.754823775 | 0.521 | 0.072 | 0 | T cells |
| LTB | 0 | 1.743987947 | 0.411 | 0.101 | 0 | T cells |
| CD8A | 0 | 1.733682199 | 0.368 | 0.057 | 0 | T cells |
| CD3E | 0 | 1.588317492 | 0.626 | 0.164 | 0 | T cells |
| CD52 | 0 | 1.510577469 | 0.74 | 0.33 | 0 | T cells |
| TRBC1 | 0 | 1.505238028 | 0.408 | 0.174 | 0 | T cells |
| CXCR4 | 0 | 1.46606891 | 0.954 | 0.6 | 0 | T cells |
| CLEC2D | 0 | 1.386347481 | 0.383 | 0.085 | 0 | T cells |
| LEPROTL1 | 0 | 1.368285415 | 0.757 | 0.435 | 0 | T cells |
| ICOS | 0 | 1.343699601 | 0.278 | 0.02 | 0 | T cells |
| CD8B | 0 | 1.330320572 | 0.251 | 0.021 | 0 | T cells |
| GNLY | 0 | 5.978457443 | 0.752 | 0.107 | 0 | Natural killer cells |
| GZMB | 0 | 3.306875475 | 0.742 | 0.101 | 0 | Natural killer cells |
| NKG7 | 0 | 3.127466854 | 0.952 | 0.295 | 0 | Natural killer cells |
| KLRC1 | 0 | 2.873180424 | 0.61 | 0.028 | 0 | Natural killer cells |
| KLRD1 | 0 | 2.67132192 | 0.827 | 0.082 | 0 | Natural killer cells |
| XCL1 | 0 | 2.608551872 | 0.542 | 0.063 | 0 | Natural killer cells |
| TRDC | 0 | 2.593126663 | 0.577 | 0.018 | 0 | Natural killer cells |
| XCL2 | 0 | 2.582824527 | 0.704 | 0.141 | 0 | Natural killer cells |
| CTSW | 0 | 2.515642325 | 0.791 | 0.166 | 0 | Natural killer cells |
| HOPX | 0 | 2.390055457 | 0.654 | 0.101 | 0 | Natural killer cells |
| IL2RB | 0 | 2.28079251 | 0.721 | 0.137 | 0 | Natural killer cells |
| KRT81 | 0 | 2.166990876 | 0.449 | 0.009 | 0 | Natural killer cells |
| CMC1 | 0 | 2.157092535 | 0.641 | 0.146 | 0 | Natural killer cells |
| KRT86 | 0 | 2.12954231 | 0.446 | 0.015 | 0 | Natural killer cells |
| PRF1 | 0 | 2.119787049 | 0.695 | 0.109 | 0 | Natural killer cells |
| CD7 | 0 | 2.074758208 | 0.846 | 0.279 | 0 | Natural killer cells |
| GZMA | 0 | 2.044074281 | 0.802 | 0.301 | 0 | Natural killer cells |
| AREG | 0 | 1.858466217 | 0.68 | 0.227 | 0 | Natural killer cells |
| KLRB1 | 0 | 1.800323281 | 0.716 | 0.167 | 0 | Natural killer cells |
| KLRC2 | 0 | 1.721313307 | 0.386 | 0.013 | 0 | Natural killer cells |
| C1QA | 0 | 4.352764996 | 0.749 | 0.013 | 0 | Macrophages |
| IL1B | 0 | 4.28989234 | 0.797 | 0.066 | 0 | Macrophages |
| C1QB | 0 | 4.116077972 | 0.712 | 0.012 | 0 | Macrophages |
| HLA-DRA | 0 | 4.115597116 | 0.967 | 0.214 | 0 | Macrophages |
| CCL3 | 0 | 4.06473441 | 0.761 | 0.173 | 0 | Macrophages |
| CCL3L1 | 0 | 4.034655909 | 0.674 | 0.105 | 0 | Macrophages |
| CXCL3 | 0 | 4.003376235 | 0.553 | 0.021 | 0 | Macrophages |
| SELENOP | 0 | 3.686447244 | 0.52 | 0.039 | 0 | Macrophages |
| CXCL2 | 0 | 3.645655561 | 0.664 | 0.057 | 0 | Macrophages |
| CST3 | 0 | 3.615586833 | 0.928 | 0.151 | 0 | Macrophages |
| CCL20 | 0 | 3.556401373 | 0.408 | 0.042 | 0 | Macrophages |
| HLA-DPA1 | 0 | 3.507610044 | 0.917 | 0.253 | 0 | Macrophages |
| RNASE1 | 0 | 3.49864869 | 0.411 | 0.023 | 0 | Macrophages |
| CD74 | 0 | 3.383825776 | 0.969 | 0.438 | 0 | Macrophages |
| C1QC | 0 | 3.306671977 | 0.678 | 0.005 | 0 | Macrophages |
| EREG | 0 | 3.285967385 | 0.36 | 0.014 | 0 | Macrophages |
| HLA-DRB1 | 0 | 3.281033732 | 0.914 | 0.249 | 0 | Macrophages |
| LYZ | 0 | 3.256560803 | 0.714 | 0.046 | 0 | Macrophages |
| CTSL | 0 | 3.222340482 | 0.701 | 0.048 | 0 | Macrophages |
| APOE | 0 | 3.176762021 | 0.471 | 0.047 | 0 | Macrophages |
| S100A9 | 0 | 5.856017784 | 0.922 | 0.073 | 0 | Neutrophils |
| S100A8 | 0 | 5.818240797 | 0.893 | 0.034 | 0 | Neutrophils |
| NAMPT | 0 | 4.207138587 | 0.972 | 0.412 | 0 | Neutrophils |
| G0S2 | 0 | 4.078410682 | 0.934 | 0.14 | 0 | Neutrophils |
| TREM1 | 0 | 4.051093997 | 0.746 | 0.061 | 0 | Neutrophils |
| FCGR3B | 0 | 4.048539669 | 0.668 | 0.002 | 0 | Neutrophils |
| CSF3R | 0 | 4.042895515 | 0.751 | 0.065 | 0 | Neutrophils |
| FPR1 | 0 | 3.904726805 | 0.758 | 0.094 | 0 | Neutrophils |
| SLC25A37 | 0 | 3.704934932 | 0.718 | 0.125 | 0 | Neutrophils |
| AQP9 | 0 | 3.485942738 | 0.648 | 0.031 | 0 | Neutrophils |
| SLC11A1 | 0 | 3.445777758 | 0.61 | 0.061 | 0 | Neutrophils |
| MNDA | 0 | 3.435217558 | 0.591 | 0.091 | 0 | Neutrophils |
| CMTM2 | 0 | 3.418428438 | 0.49 | 0.001 | 0 | Neutrophils |
| C5AR1 | 0 | 3.390569054 | 0.808 | 0.17 | 0 | Neutrophils |
| CLEC4E | 0 | 3.315315607 | 0.544 | 0.048 | 0 | Neutrophils |
| BCL2A1 | 0 | 3.250340538 | 0.879 | 0.254 | 0 | Neutrophils |
| LUCAT1 | 0 | 3.228416192 | 0.61 | 0.085 | 0 | Neutrophils |
| MXD1 | 0 | 3.224573704 | 0.728 | 0.198 | 0 | Neutrophils |
| BASP1 | 0 | 3.215300165 | 0.754 | 0.173 | 0 | Neutrophils |
| IFITM2 | 0 | 3.175630315 | 0.952 | 0.726 | 0 | Neutrophils |
| TPSB2 | 0 | 7.145650488 | 0.967 | 0.008 | 0 | Mast cells |
| TPSAB1 | 0 | 6.449720471 | 0.956 | 0.004 | 0 | Mast cells |
| CPA3 | 0 | 5.412318096 | 0.936 | 0.001 | 0 | Mast cells |
| CTSG | 0 | 4.000721644 | 0.473 | 0.001 | 0 | Mast cells |
| MS4A2 | 0 | 3.819810842 | 0.849 | 0.001 | 0 | Mast cells |
| HPGDS | 0 | 3.278627107 | 0.831 | 0.051 | 0 | Mast cells |
| IL1RL1 | 0 | 3.048040831 | 0.762 | 0.007 | 0 | Mast cells |
| HPGD | 0 | 2.948128856 | 0.704 | 0.094 | 0 | Mast cells |
| CD9 | 0 | 2.934223381 | 0.868 | 0.162 | 0 | Mast cells |
| CLU | 0 | 2.899388518 | 0.822 | 0.07 | 0 | Mast cells |
| GATA2 | 0 | 2.795624638 | 0.659 | 0.021 | 0 | Mast cells |
| LMNA | 0 | 2.784242016 | 0.921 | 0.476 | 0 | Mast cells |
| SLC18A2 | 0 | 2.713480072 | 0.712 | 0.009 | 0 | Mast cells |
| KIT | 0 | 2.624823932 | 0.696 | 0.017 | 0 | Mast cells |
| HDC | 0 | 2.562538533 | 0.655 | 0.001 | 0 | Mast cells |
| VWA5A | 0 | 2.538193747 | 0.672 | 0.009 | 0 | Mast cells |
| RHEX | 0 | 2.494494794 | 0.638 | 0.007 | 0 | Mast cells |
| LTC4S | 0 | 2.297557964 | 0.592 | 0.071 | 0 | Mast cells |
| CALB2 | 0 | 2.04046819 | 0.565 | 0.005 | 0 | Mast cells |
| TUBA1A | 4.80E-209 | 2.325124032 | 0.78 | 0.443 | 1.30E-204 | Mast cells |
| JCHAIN | 0 | 3.930299449 | 0.844 | 0.01 | 0 | Plasmacytoid dendritic cells |
| IL3RA | 0 | 2.507122365 | 0.731 | 0.035 | 0 | Plasmacytoid dendritic cells |
| EGLN3 | 0 | 2.502779916 | 0.731 | 0.01 | 0 | Plasmacytoid dendritic cells |
| PLD4 | 0 | 2.43652378 | 0.772 | 0.06 | 0 | Plasmacytoid dendritic cells |
| OPN3 | 1.36E-302 | 2.498239012 | 0.689 | 0.051 | 3.68E-298 | Plasmacytoid dendritic cells |
| IRF4 | 2.06E-237 | 3.382788918 | 0.88 | 0.117 | 5.58E-233 | Plasmacytoid dendritic cells |
| SOX4 | 6.13E-170 | 3.728264711 | 0.844 | 0.148 | 1.66E-165 | Plasmacytoid dendritic cells |
| C12orf75 | 2.38E-169 | 2.599088655 | 0.838 | 0.144 | 6.44E-165 | Plasmacytoid dendritic cells |
| TCF4 | 2.47E-164 | 2.618997543 | 0.844 | 0.151 | 6.71E-160 | Plasmacytoid dendritic cells |
| PPP1R14B | 2.18E-146 | 2.464046166 | 0.862 | 0.176 | 5.91E-142 | Plasmacytoid dendritic cells |
| GPR183 | 1.40E-111 | 3.557690731 | 0.97 | 0.418 | 3.80E-107 | Plasmacytoid dendritic cells |
| ETV3 | 5.45E-107 | 2.516888501 | 0.772 | 0.183 | 1.48E-102 | Plasmacytoid dendritic cells |
| RRBP1 | 9.45E-106 | 2.329998494 | 0.844 | 0.238 | 2.56E-101 | Plasmacytoid dendritic cells |
| NR4A3 | 2.39E-101 | 2.364921029 | 0.928 | 0.319 | 6.47E-97 | Plasmacytoid dendritic cells |
| LDLRAD4 | 9.62E-88 | 2.275907602 | 0.844 | 0.273 | 2.61E-83 | Plasmacytoid dendritic cells |
| DSTN | 1.94E-83 | 2.260441315 | 0.904 | 0.346 | 5.26E-79 | Plasmacytoid dendritic cells |
| SEC61B | 3.80E-82 | 2.374448357 | 0.958 | 0.555 | 1.03E-77 | Plasmacytoid dendritic cells |
| GZMB | 1.89E-81 | 2.313880033 | 0.844 | 0.257 | 5.11E-77 | Plasmacytoid dendritic cells |
| AREG | 8.33E-65 | 2.32011483 | 0.856 | 0.337 | 2.26E-60 | Plasmacytoid dendritic cells |
| PTGDS | 1.93E-40 | 2.935658902 | 0.407 | 0.108 | 5.24E-36 | Plasmacytoid dendritic cells |

# Table S7. Top 20 differentially expressed genes of mural cell subclusters

| **Gene Name** | **p_val** | **avg_log2FC** | **pct.1** | **pct.2** | **p_val_adj** | **C ell Type** |
| --- | --- | --- | --- | --- | --- | --- |
| STEAP4 | 0 | 2.076604656 | 0.458 | 0.039 | 0 | Prv_STEAP4 |
| CTSC | 0 | 1.906471434 | 0.668 | 0.244 | 0 | Prv_STEAP4 |
| COL4A1 | 0 | 1.658224175 | 0.755 | 0.561 | 0 | Prv_STEAP4 |
| RGS5 | 0 | 1.658148403 | 0.815 | 0.535 | 0 | Prv_STEAP4 |
| GUCY1A2 | 0 | 1.559881338 | 0.737 | 0.359 | 0 | Prv_STEAP4 |
| COL1A2 | 0 | 1.529182845 | 0.863 | 0.678 | 0 | Prv_STEAP4 |
| CYTOR | 0 | 1.528514241 | 0.594 | 0.352 | 0 | Prv_STEAP4 |
| TCIM | 0 | 1.465874647 | 0.54 | 0.145 | 0 | Prv_STEAP4 |
| HIGD1B | 0 | 1.459238486 | 0.663 | 0.287 | 0 | Prv_STEAP4 |
| THY1 | 0 | 1.440745074 | 0.546 | 0.212 | 0 | Prv_STEAP4 |
| SPARC | 0 | 1.411285924 | 0.921 | 0.864 | 0 | Prv_STEAP4 |
| NRP1 | 0 | 1.379947622 | 0.632 | 0.285 | 0 | Prv_STEAP4 |
| GGT5 | 0 | 1.373718558 | 0.436 | 0.055 | 0 | Prv_STEAP4 |
| EDNRB | 0 | 1.346084991 | 0.381 | 0.071 | 0 | Prv_STEAP4 |
| IFITM1 | 0 | 1.321472205 | 0.491 | 0.142 | 0 | Prv_STEAP4 |
| COL6A3 | 0 | 1.317639044 | 0.604 | 0.296 | 0 | Prv_STEAP4 |
| COL1A1 | 0 | 1.3018754 | 0.83 | 0.686 | 0 | Prv_STEAP4 |
| COL3A1 | 0 | 1.300721259 | 0.872 | 0.682 | 0 | Prv_STEAP4 |
| ADGRF5 | 0 | 1.297011527 | 0.386 | 0.038 | 0 | Prv_STEAP4 |
| CCL2 | 3.55E-175 | 1.343063965 | 0.568 | 0.45 | 9.62E-171 | Prv_STEAP4 |
| RERGL | 0 | 2.796218259 | 0.821 | 0.07 | 0 | VSMC |
| MT1X | 0 | 2.640893032 | 0.821 | 0.356 | 0 | VSMC |
| ADIRF | 0 | 2.334066647 | 0.994 | 0.633 | 0 | VSMC |
| MUSTN1 | 0 | 2.296532261 | 0.799 | 0.297 | 0 | VSMC |
| PLN | 0 | 2.105137581 | 0.86 | 0.226 | 0 | VSMC |
| MT1M | 0 | 2.037112007 | 0.882 | 0.375 | 0 | VSMC |
| CRIP1 | 0 | 1.988827123 | 0.974 | 0.595 | 0 | VSMC |
| PTN | 0 | 1.978253695 | 0.906 | 0.423 | 0 | VSMC |
| SNCG | 0 | 1.947238713 | 0.693 | 0.123 | 0 | VSMC |
| GADD45B | 0 | 1.906989864 | 0.959 | 0.793 | 0 | VSMC |
| SORBS2 | 0 | 1.841638939 | 0.689 | 0.08 | 0 | VSMC |
| DSTN | 0 | 1.838549375 | 0.991 | 0.848 | 0 | VSMC |
| S100A4 | 0 | 1.726034367 | 0.923 | 0.478 | 0 | VSMC |
| KLF2 | 0 | 1.661786195 | 0.687 | 0.328 | 0 | VSMC |
| MT1E | 0 | 1.646502227 | 0.868 | 0.439 | 0 | VSMC |
| BCAM | 0 | 1.63826585 | 0.855 | 0.317 | 0 | VSMC |
| NDUFA4 | 0 | 1.577820106 | 0.936 | 0.575 | 0 | VSMC |
| C12orf75 | 0 | 1.530673712 | 0.518 | 0.13 | 0 | VSMC |
| MYH11 | 0 | 1.50538168 | 0.969 | 0.501 | 0 | VSMC |
| NTRK2 | 0 | 1.497577976 | 0.737 | 0.111 | 0 | VSMC |
| ACTG2 | 0 | 4.480574039 | 0.913 | 0.258 | 0 | uSMC |
| DES | 0 | 3.68952077 | 0.899 | 0.175 | 0 | uSMC |
| CNN1 | 0 | 2.970762909 | 0.942 | 0.424 | 0 | uSMC |
| SFRP4 | 0 | 2.383392896 | 0.848 | 0.284 | 0 | uSMC |
| MFAP5 | 0 | 2.290631492 | 0.523 | 0.012 | 0 | uSMC |
| MYLK | 0 | 2.228578368 | 0.963 | 0.77 | 0 | uSMC |
| CSRP1 | 0 | 2.198303621 | 0.916 | 0.586 | 0 | uSMC |
| RAMP1 | 0 | 2.146276277 | 0.953 | 0.47 | 0 | uSMC |
| TPM1 | 0 | 1.925336309 | 0.983 | 0.837 | 0 | uSMC |
| SLMAP | 0 | 1.91065502 | 0.773 | 0.192 | 0 | uSMC |
| SMOC2 | 0 | 1.904796365 | 0.831 | 0.13 | 0 | uSMC |
| IGFBP2 | 0 | 1.865495857 | 0.719 | 0.349 | 0 | uSMC |
| TCEAL4 | 0 | 1.831302819 | 0.969 | 0.653 | 0 | uSMC |
| SFRP1 | 0 | 1.826939182 | 0.63 | 0.149 | 0 | uSMC |
| LPP | 0 | 1.786961436 | 0.95 | 0.626 | 0 | uSMC |
| CDC42EP3 | 0 | 1.702036572 | 0.74 | 0.068 | 0 | uSMC |
| TAGLN | 0 | 1.688843385 | 0.988 | 0.906 | 0 | uSMC |
| KCNMA1 | 0 | 1.687478404 | 0.769 | 0.13 | 0 | uSMC |
| TPM2 | 0 | 1.663675024 | 0.99 | 0.948 | 0 | uSMC |
| ATP1B1 | 0 | 1.661114062 | 0.742 | 0.089 | 0 | uSMC |
| SFRP4 | 0 | 3.021213606 | 0.902 | 0.287 | 0 | Myofibroblast |
| SFRP1 | 0 | 2.563097086 | 0.76 | 0.143 | 0 | Myofibroblast |
| IGF1 | 0 | 1.931520307 | 0.787 | 0.219 | 0 | Myofibroblast |
| DCN | 0 | 1.899513613 | 0.904 | 0.347 | 0 | Myofibroblast |
| ECM1 | 0 | 1.757899947 | 0.698 | 0.177 | 0 | Myofibroblast |
| MMP11 | 0 | 1.75125741 | 0.546 | 0.181 | 0 | Myofibroblast |
| PTGDS | 0 | 1.656285726 | 0.717 | 0.176 | 0 | Myofibroblast |
| VCAN | 0 | 1.598814978 | 0.732 | 0.193 | 0 | Myofibroblast |
| MEG3 | 0 | 1.571953447 | 0.695 | 0.173 | 0 | Myofibroblast |
| MME | 0 | 1.550746107 | 0.509 | 0.035 | 0 | Myofibroblast |
| ENPP2 | 0 | 1.513626399 | 0.456 | 0.086 | 0 | Myofibroblast |
| MDK | 0 | 1.50196221 | 0.931 | 0.478 | 0 | Myofibroblast |
| LUM | 0 | 1.456839996 | 0.775 | 0.244 | 0 | Myofibroblast |
| MMP2 | 0 | 1.446160964 | 0.731 | 0.157 | 0 | Myofibroblast |
| CD24 | 0 | 1.396506901 | 0.624 | 0.125 | 0 | Myofibroblast |
| IGFBP2 | 0 | 1.376326357 | 0.75 | 0.351 | 0 | Myofibroblast |
| PGRMC1 | 0 | 1.375147794 | 0.748 | 0.366 | 0 | Myofibroblast |
| PAGE4 | 0 | 1.374999189 | 0.317 | 0.039 | 0 | Myofibroblast |
| ITM2B | 0 | 1.343768767 | 0.954 | 0.824 | 0 | Myofibroblast |
| RORB | 0 | 1.320350094 | 0.587 | 0.11 | 0 | Myofibroblast |

# Table S8. Sequences of qPCR primers

| **Gene** | **Forward 5'-3'** | **Reverse 5'-3'** |
| --- | --- | --- |
| *IFNγ* | TGAATGTCCAACGCAAAGCAA | ACTGGGATGCTCTTCGACCT |
| *IFNα* | GGATGAGGACCTCCTAGACAA | GGGAGTTTCTCCCACCCTCT |
| *IL1β* | ATGATGGCTTATTACAGTGGCAA | GTCGGAGATTCGTAGCTGGA |
| *IL-17A* | CGGACTGTGATGGTCAACCTGA | GCACTTTGCCTCCCAGATCACA |
| *GAPDH* | GGAGCGAGATCCCTCCAAAAT | GGCTGTTGTCATACTTCTCATGG |
